# Single-cell sequencing reveals the landscape of the tumor microenvironment in a skeletal undifferentiated pleomorphic sarcoma patient

**DOI:** 10.3389/fimmu.2022.1019870

**Published:** 2022-11-16

**Authors:** Liu-Liu Yuan, Zhong Chen, Jian Qin, Cheng-Jiao Qin, Jing Bian, Rui-Fang Dong, Tang-Bo Yuan, Yi-Ting Xu, Ling-Yi Kong, Yuan-Zheng Xia

**Affiliations:** ^1^ Jiangsu Key Laboratory of Bioactive Natural Product Research and State Key Laboratory of Natural Medicines, School of Traditional Chinese Pharmacy, China Pharmaceutical University, Nanjing, China; ^2^ Department of Orthopedics, Sir Run Run Hospital, Nanjing Medical University, Nanjing, China; ^3^ Key Laboratory of Early Prevention and Treatment for Regional High Frequency Tumor (Guangxi Medical University), Ministry of Education and Guangxi Key Laboratory of Early Prevention and Treatment for Regional High Frequency Tumor, Nanning, China

**Keywords:** single-cell RNA sequencing, skeletal undifferentiated pleomorphic sarcoma, tirelizumab, PD-1, T cells

## Abstract

Skeletal undifferentiated pleomorphic sarcoma (SUPS) is an invasive pleomorphic soft tissue sarcoma with a high degree of malignancy and poor prognosis. It is prone to recur and metastasize. The tumor microenvironment (TME) and the pathophysiology of SUPS are barely described. Single-cell RNA sequencing (scRNA-seq) provides an opportunity to dissect the landscape of human diseases at an unprecedented resolution, particularly in diseases lacking animal models, such as SUPS. We performed scRNA-seq to analyze tumor tissues and paracancer tissues from a SUPS patient. We identified the cell types and the corresponding marker genes in this SUPS case. We further showed that CD8^+^ exhausted T cells and Tregs highly expressed *PDCD1*, *CTLA4* and *TIGIT*. Thus, *PDCD1*, *CTLA4* and *TIGIT* were identified as potential targets in this case. We applied copy number karyotyping of aneuploid tumors (CopyKAT) to distinguish malignant cells from normal cells in fibroblasts. Our study identified eight malignant fibroblast subsets in SUPS with distinct gene expression profiles. C1-malignant Fibroblast and C6-malignant Fibroblast in the TME play crucial roles in tumor growth, angiogenesis, metastasis and immune response. Hence, targeting malignant fibroblasts could represent a potential strategy for this SUPS therapy. Intervention *via* tirelizumab enabled disease control, and immune checkpoint inhibitors (ICIs) of PD-1 may be considered as the first-line option in patients with SUPS. Taken together, scRNA-seq analyses provided a powerful basis for this SUPS treatment, improved our understanding of complex human diseases, and may afforded an alternative approach for personalized medicine in the future.

## Introduction

The most common term for a generic high-grade sarcoma has evolved over the years from fibrosarcoma to malignant fibrous histiocytoma (MFH) and now to high-grade undifferentiated pleomorphic sarcoma (UPS), as of the writing of 2013 WHO sarcoma classification system. The term UPS of bone is increasingly used instead of MFH of bone (1–3). Skeletal undifferentiated pleomorphic sarcoma (SUPS) is a matrix-producing malignant tumor with a pleomorphic spindle-cell structure, which is devoid of any specific pattern of histologic differentiation (4). SUPS often exhibit aggressive behavior associated with high metastatic potential and a high rate of local recurrence (5). Bone neoplasms are rare solid tumors, accounting for less than 2% of all primary malignancies. SUPS is an extremely rare and aggressive malignancy representing <1% of all primary malignant bone tumors (6). SUPS often occurs in the bone diaphysis or metaphysis and results in invasive bone damage and a soft tissue mass. The pathophysiology remains elusive, and its therapeutic options are limited (7, 8). Here, we report a rare case of SUPS at the lower femoral end of the left thigh in a 44-year-old man. However, current knowledge of SUPS is limited to case reports and small case series. The clinicopathological features and prognosis, tumor microenvironment (TME) and tumor heterogeneity of these cancers have not been well defined.

Over the past ten years, the rapid development of single-cell RNA sequencing (scRNA-seq) has enabled us to quickly obtain a large amount of physiological and pathological information on various tumors (9). We hypothesized that the scRNA-seq approach to determine single-cell transcriptomic changes might provide a powerful personalized medicine tool that not only deepens our insight into disease mechanisms but also enables the identification of overexpressed genes or altered pathways that might be targeted *via* currently available monoclonal antibodies or small-molecule inhibitors (10–12). Notably, scRNA-seq has been widely used to reveal the characteristics of immunity in various fields because it can detect changes in individual cell types. The emergence of single-cell sequencing represents a powerful tool to resolve tumor heterogeneity and delineate the complex communication among tumor cells with neighboring stromal and immune cells in the TME (13). Paracancer tissues are commonly used as a control in cancer studies (14). However, an increasing number of studies have shown that paracancer tissues are at the transition between cancer and normal tissues, and the expression of key molecules and the microenvironment have been changed (15). However, the critical roles of paracancer tissues in cancer research have not received enough attention. Here, we performed scRNA-seq on the paracancer tissues and tumor tissues from this SUPS patient. These unprecedented data uncovered the transcriptional landscape and phenotypic heterogeneity of tumor and immune cells in SUPS, and identified their gene expression signature, suggesting specialized functions.

Targeting specific immunological pathways represents a promising approach to fighting tumors (16). T-cell exhaustion was indicated by multiple inhibitory receptors, and we found that CD8-C1-*PDCD1* (CD8^+^ exhausted T cells) and CD4-C2-*FOXP3* (CD4^+^ Tregs) in both tissues positively expressed T-cell inhibitory receptors, including *PDCD1* and *CTLA4*. At present, inhibitors corresponding to programmed death protein-1 (PD-1) and CTLA4 are widely used in clinical practice. Our study provided evidence to the attending physician that this patient would benefit from administrating inhibitors targeting one or both of these factors. There are no guidelines for the employment of immune checkpoint inhibitors (ICIs) in SUPS therapy, and treatment outcomes have rarely been reported. We report the first case of SUPS with PD-1 ICI administration, describing the clinical features, imaging, pathological findings, and TME. In this case, the successful intervention yielded outcomes superior to those of previous patients with SUPS. ICIs of PD-1 may be considered the first-line therapy for patients with SUPS.

## Results

### Imaging examinations and further diagnostic work-up of SUPS

**Figure 1:**
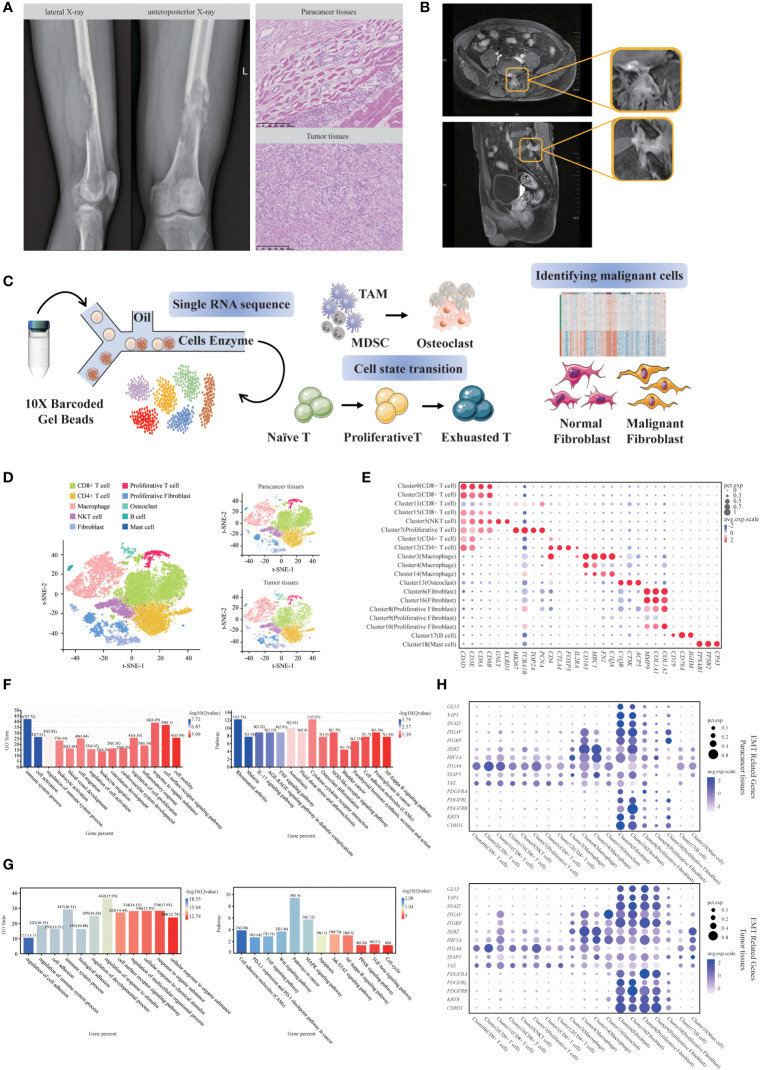
Single-cell transcriptomic analysis of SUPS. **(A)** Bone destruction of the left lower femur with a soft tissue mass (Lateral X-ray film; Anteroposterior X-ray). H&E staining of the tumor tissues and paracancer tissues (Scale bar 250μm). **(B)** MR medical impact (upper: pelvic plain scan; lower: femoral plain scan). **(C)** Schematic diagram of the experimental design. **(D)** The t-distributed stochastic neighbor embedding (t-SNE) plot of the 10 identified main cell types in SUPS lesions, with each cell color-coded according to its associated cell type. **(E)** Dot plot showing the 30 signature gene expressions across the 10 cellular clusters. The size of dots represents the proportion of cells expressing the particular marker, and the spectrum of color indicates the mean expression levels of the markers (log1p transformed). **(F)** GO and KEGG enrichment analyses were performed for DEGs in SUPS between tumor tissues and paracancer tissues. Representative significantly enriched function processes are shown. **(G)** GO and KEGG enrichment analyses were performed for DEGs in non-immune cells between tumor tissues and paracancer tissues. **(H)** Expression of EMT-related genes were shown for each cluster of paracancer tissues and tumor tissues. Dot size corresponded to the percentage of cells in the cluster expressing a gene, and dot color corresponded to the average expression level for the gene in the cluster.

Here, we report a rare case of SUPS in the lower femoral segment of the left thigh in a 44-year-old male patient. In February 2021, the patient complained of pain and discomfort in the distal left thigh and went to a local hospital for symptomatic conservative treatment. The result of treatment in the local hospital was not good. The patient suffered from severe pain and found a hard mass at the distal end of his left thigh for one month. Therefore, he came to our hospital for further diagnosis and treatment. X-ray showed irregular osteolytic bone destruction at the lower end of the left femur, which invaded the bone marrow cavity. Multiple strip-like high-density dead bone shadows were seen inside (**Figure 1A**). CT and MRI showed a mass of soft tissue at the lower end of the left femur and its surrounding region (**Supplementary Figure S1A**). Hematoxylin-eosin (H&E) staining showed spindle cells clustered in the damaged area of bone (**Figure 1A**). Immunohistochemical staining showed SMA (+), CD68 (+), Ki67 (+), CD34 (+), Desmin (-), EMA (-), S-100 (-) and Bcl-2 (-) (**Supplementary Figure S1B**). The pathological result of this patient was SUPS (8). To reduce the burden on the tumor and relieve the symptoms of leg pain, a tumor resection was performed to remove the tumor on June 24th, 2021.

Two months later, the results of the patient’s MRI showed a patchy abnormal signal on the left side of the L5 spinous process and the posterior edge of the vertebral body, which was slightly more advanced than before, and the enhancement was slightly increased. The abnormal signal at the left posterior border of the sacrum was similar to the anterior range and slightly reduced in the signal. The patient’s pelvic pain scan on August 20th, 2021 showed an abnormal imaging signal around the left iliac vessels and left inguinal region, considering lymph node enlargement. The possibility of metastasis was considered based on these results and medical history (**Figure 1B**). We performed scRNA-seq on tumor tissues and paracancer tissues from this patient after the surgery. We discovered that CD8+ exhausted T cells and Tregs highly expressed *PDCD1*, *CTLA4* and *TIGIT*. Subsequently, we identified *PDCD1*, *CTLA4* and *TIGIT* as potential targets in this case. Based on these results and the scRNA-seq analyses, we recommend that physicians use anti-CTLA4 drugs and anti-PD-1 drugs in combination. On August 25th, 2021, due to the outbreak in Nanjing and personal economic reasons, the patient underwent a second round of chemotherapy in Nanjing Gaochun People’s Hospital. The patient was treated with the anti-PD-1 drug tirelizumab in his second to fifth rounds of chemotherapy. The first chemotherapy regimen was epirubicin hydrochloride injection (100 mg, day 1) and ifosfamide injection (2 g, days 1-5). The second to fifth chemotherapy regimens were as follows: epirubicin hydrochloride injection (100 mg, day 1), ifosfamide injection (2 g, days 1-5), tislelizumab injection (200 mg, day 1), and anlotinib (10 mg, days 1-5). On November 11th, 2021, the patient was reexamined with routine blood tests. The examination of tumor markers showed that levels of alpha-fetoprotein, carcinoembryonic antigen, carbohydrate antigen 19-9 and carbohydrate antigen 12-5 returned to normal, the metastases disappeared, and no new metastases were found. Currently, the condition of the patient is stable. Thus, scRNA-seq analyses provided a successful therapeutic basis for the SUPS patient.

### scRNA-seq identified SUPS-associated cellular components in tumor tissues and paracancer tissues

To assess altered gene expression in SUPS, we performed scRNA-seq of this patient referred to the Sir Run Run Hospital Nanjing Medical University. For this, we dissociated tumor tissues into a single-cell suspension and performed scRNA-seq analysis. Paracancer tissues served as controls. The harvested cells from different groups were sequenced on a 10 × Genomics platform (**Figure 1C**), and we obtained a total of 18433 single-cell transcriptomes (10532 paracancer tissues; 7901 tumor tissues) from the two samples. We conducted preliminary quality control and evaluation of the sequencing results, removed reads with low sequencing quality, mapped reads with the reference genome using CellRanger, annotated reads as specific genes, corrected unique molecular identifiers (UMIs), and counted them (**Supplementary Figure S2**). Unbiased clustering of the cells identified 19 clusters in parallel, based on t-distributed stochastic neighbor embedding (t-SNE) and uniform manifold approximation and projection (UMAP) analyses according to their gene profiles and canonical markers (17–20) (**Figure 1D**; **Supplementary Figure S3A**). Our initial goal was to visualize and ultimately define the various cell subsets (12, 21, 22) in the dataset; these subsets included CD8^+^ T cells (4 cell clusters), CD4^+^ T cells (2 cell clusters), NKT cells (1 cell cluster), Proliferative T cells (1 cell cluster), Macrophages (3 cell clusters), Osteoclasts (1 cell cluster), Fibroblasts (2 cell clusters), Proliferative Fibroblasts (3 cell clusters), B cells (1 cell cluster) and Mast cells (1 cell cluster). In particular, we identified the marker genes for each cluster as follows: (1) CD4^+^ T cells highly express *CD4* but express *CD8* at a low level; (2) CD8^+^ T cells highly express *CD8* but express *CD4* at a low level; (3) NKT cells highly express NK-cell markers and T-cell markers *GNLY* and *GZMB*; (4) Proliferative T cells highly express T-cell markers and the proliferation markers *TUBA1B* and *MKI67*; (5) Macrophages have high expression of the markers *C1QC* and *C1QA*; (6) Osteoclasts specifically express the markers *CTSK* and *MMP9*; (7) Fibroblasts express *COL1A1* and *FN1*; (8) Proliferative Fibroblasts have high expression of proliferative markers and fibroblast markers; (9) B cells specifically express *IGHM* and *CD79A*; and (10) Mast cells highly express *TPSAB1* and *TPSB2* (**Figure 1E**; **Supplementary Figure S3B**). We calculated the correlation between cell subsets and generated a heatmap. The two cell subsets with high correlation in the figure have relatively similar gene expression patterns, indicating that they may be the same cell type (**Supplementary Figure S3C**). Subsequently, Gene Ontology (GO) enrichment analysis and Kyoto Encyclopedia of Genes and Genomes (KEGG) pathway analysis of the upregulated genes identified specific processes relevant to each cell type (**Supplementary Figure S4**).

Next, we attempted to discern the cellular differences between tumor tissues and paracancer tissues. We noticed that almost all types of cell populations were present in both tumor tissues and paracancer tissues; however, some fibroblasts were almost exclusively observed in paracancer tissues, and other were predominantly observed in the tumor tissues (**Figure 1D**). Each cell subset contained a variable number of cells and variable transcriptional activity determined by UMIs. We detected the relative abundance of infiltrating immune cells in tumor tissues and paracancer tissues, which first revealed the landscape of infiltrating immune cells in SUPS (**Supplementary Figure S3D-F**), contributing to the improvement of SUPS immunotherapy (23, 24).

GO and KEGG analyses of the differentially expressed genes (DEGs) in tumor tissues and paracancer tissues revealed that DEGs were enriched in different biological processes and indicated that SUPS is related to the regulation of immune system processes, cell activation, blood vessel development, cell migration and cell motility (**Figure 1F**). We found numerous distinctions in the gene expression within the clusters, suggesting that the biological features of tumor tissues differed from those of paracancer tissues, especially in terms of nonimmune cells (**Supplementary Figure S3G**). GO and KEGG analyses of the DEGs of nonimmune cells in tumor tissues and paracancer tissues revealed that the DEGs were enriched in pathways in cancer (**Figure 1G**). The scRNA-seq data were used to quantify the expression of genes associated with disease developmental pathways, including TGF-β, MAPK, NF-κB and JAK-STAT, as well as signaling pathways associated with epithelial-to-mesenchymal transition (EMT), in various SUPS cell populations (**Supplementary Figure S5**; **Figure 1H**). Some genes related to the MAPK, NF-κB and JAK-STAT pathways were upregulated in Osteoclasts and Fibroblasts within SUPS lesions, but few changes were found in immune cells. The EMT process has been indicated to play an important role in cancer invasion, metastasis and drug resistance. The analysis of gene signatures associated with EMT programming showed that EMT markers were significantly highly expressed in Osteoclasts and Fibroblasts (25), suggesting that most Osteoclasts and Fibroblasts in this sample were undergoing an active EMT process. Interestingly, most immune cells were enriched for fewer EMT-related genes, but Macrophages showed high enrichment of EMT-related genes (**Supplementary Figure S5**; **Figure 1H**).

Consistent with the high degree of EMT, the SUPS sample also showed significantly high levels of invasion, metastasis and angiogenesis according to gene signature scores (**Supplementary Figure S3H**), indicating that the SUPS in this patient might have an increased capability for high-grade metastasis, which highly correlates with a poor prognosis (11). Indeed, this patient presented with metastasis based on clinical examination approximately two months after the surgery. SUPS exhibits a high number of tumor-infiltrating immune cells (TIICs), suggesting that SUPS could benefit from immune checkpoint blockade (ICB). However, not all SUPS patients respond to neoadjuvant ICB. Thus, we need to identify which underlying mechanisms and associated markers determine therapeutic response. TIICs represent a heterogeneous population of cells concerning cell type composition, gene expression and functional properties. To date, TIIC scores and tumor PD-1 expression have been proposed to predict clinical outcomes, but their ability to act as predictors for SUPS remains unclear (26, 27).

### Gene expression heterogeneity of T-cell subsets was identified in the SUPS patient

**Figure 2:**
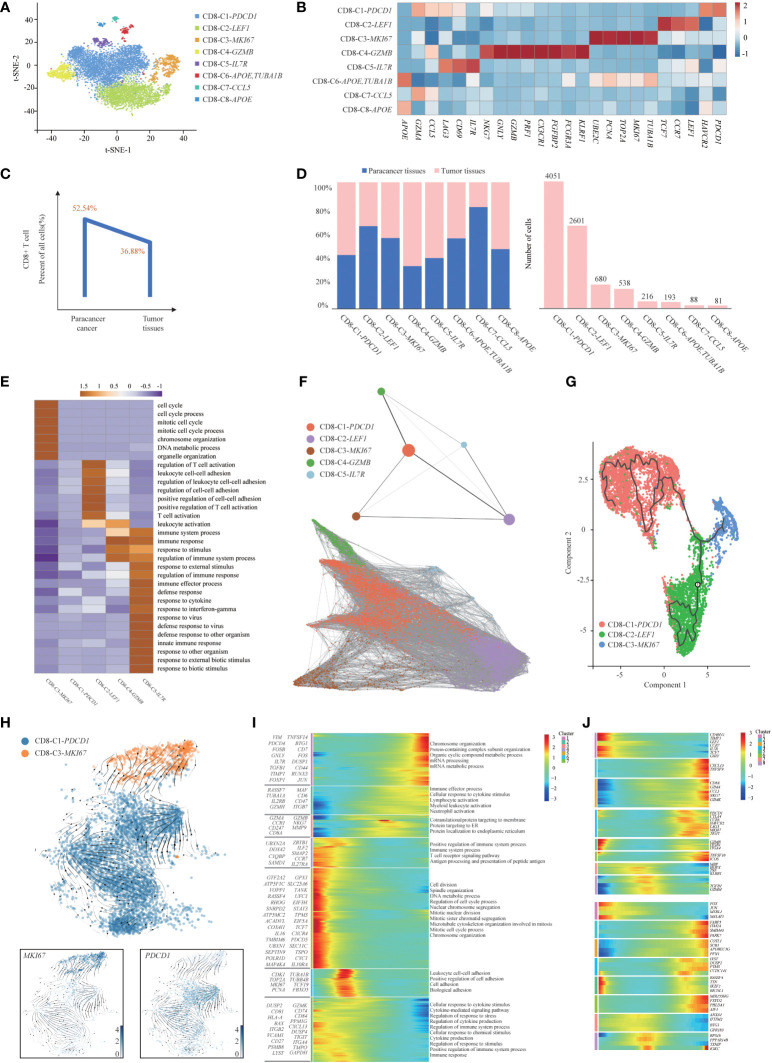
Gene expression heterogeneity of CD8^+^ T cell subsets was identified in the SUPS. **(A)** t-SNE plot showing the eight main subsets of CD8^+^ T cells. **(B)** Relative expression map of known marker genes associated with each cell subset. Mean expression values are scaled by mean-centering, and transformed to a scale from -1 to 2. **(C)**. The percentages of CD8^+^ T cells in paracancer tissues and tumor tissues. **(D)** The cell number and proportion of each CD8^+^ T cell cluster. **(E)** Functional enrichment analysis of upregulated genes in each CD8^+^ T cells cluster was performed with GO analysis. Representative significantly enriched function processes are shown. **(F)** Pseudotime trajectories for CD8^+^ T cells (CD8-C1-*PDCD1*, CD8-C2-*LEF1*, CD8-C3-*MKI67*, CD8-C4-*GZMB*, CD8-C5-*IL7R*) based on PAGA. **(G)** The Monocle 3 trajectory plot showed the dynamics of CD8-C1-*PDCD1*, CD8-C2-*LEF1* and CD8-C3-*MKI67*. **(H)** RNA velocities are visualized on the UMAP projection of CD8-C3-*MKI67* and CD8-C1-*PDCD1* populations, colored by clusters. **(I)** The DEGs (in rows, *q*-value < 10^−10^) in CD8^+^ T cells along the pseudotime were hierarchically clustered into different subsets. The top annotated GO terms in each cluster were provided. **(J)** Gene expression dynamics along the CD8-C1-*PDCD1* trajectory. Genes cluster into different gene sets, each characterized by specific expression profiles (upper). For each gene cluster (indicated by different colors), the expression of some novel genes along the trajectory is shown (lower).

**Figure 3:**
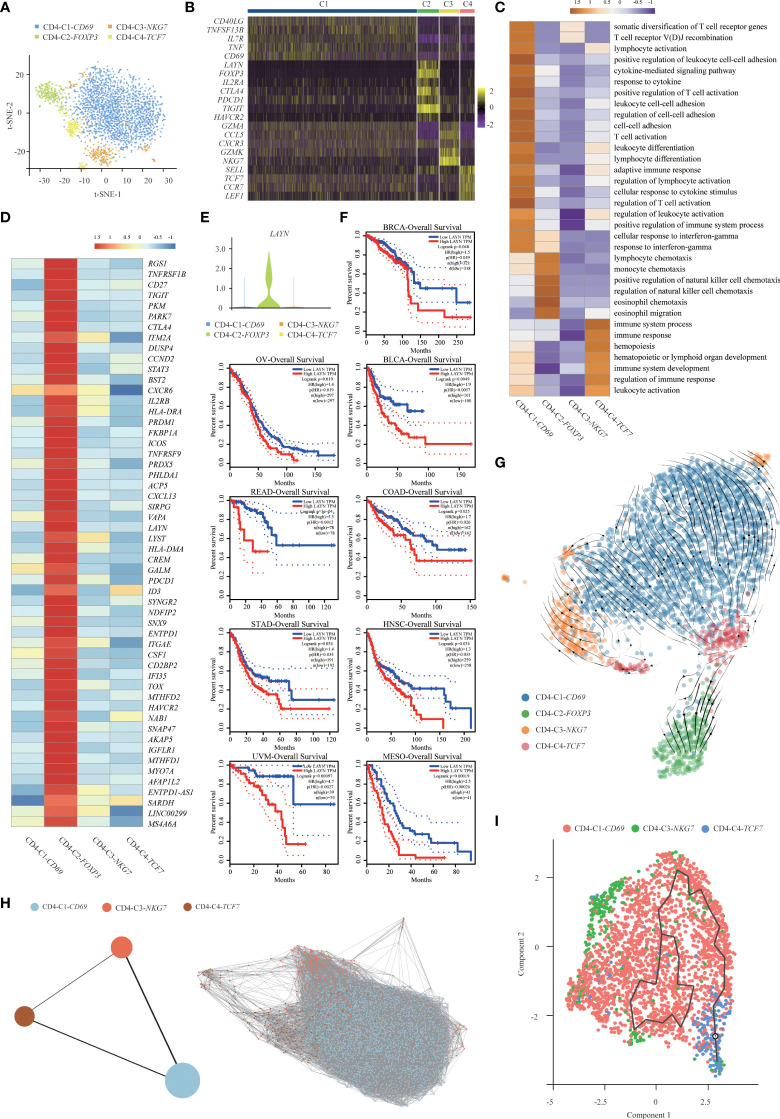
Gene expression heterogeneity of CD4^+^ T cell subsets was identified in the SUPS. **(A)** t-SNE plot showing the four main subsets of CD4^+^ T cells. **(B)** Heatmap showing specific marker genes in each CD4^+^ T cells cluster (each column represents an individual cluster; purple represents the minimum, black represents the median, and yellow represents the maximum expression values). **(C)** Functional enrichment analysis of upregulated genes in each CD4^+^ T cells cluster was performed with GO analysis. **(D)** Heatmap comparing the expression of exhaustion-related genes in CD4^+^ T cells cluster. **(E)** Violin plot comparing the expression of *LAYN* in CD4^+^ T cells cluster. **(F)** The disease-free survival curve based on TCGA data showed patients with higher expression of *LAYN* had poor prognoses. **(G)** RNA velocities are visualized on the UMAP projection of CD4^+^ T cell populations, colored by clusters. **(H)** Pseudotime trajectories for CD4^+^ T cells (CD4-C1-*CD69*, CD4-C3-*NKG7*, CD4-C4-*TCF7*) based on PAGA. **(I)** The Monocle 3 trajectory plot shows the dynamics of CD4-C1-*CD69*, CD4-C3-*NKG7* and CD4-C4-*TCF7*.

T cells are the key elements of cancer immunotherapy. However, their high heterogeneity regarding their cell-type compositions, gene expression patterns and functional properties significantly influence the outcomes of T-cell-based immunotherapy. Interestingly, we found that T-cell clusters were present at high levels in immune cells (**Figure 1D**), and the presence of infiltrating T cells in tumor tissues was confirmed using immunohistochemistry (IHC) with CD3E, CD4 and CD8 antibodies. IHC analyses were consistent with the results of the scRNA-seq data (**Supplementary Figure S6A**), thus demonstrating that T-cell-based immunotherapy might be efficient in this SUPS patient. Notably, we observed that the overall number of T cells in tumor tissues was much lower than that in the corresponding paracancer tissues (**Figure 1D**; **Supplementary Figure S6B**), indicating that T-cell infiltration was inefficient (21). There are many differences in gene expression within T-cell clusters, suggesting that T-cell biology in tumor tissues differs from that in paracancer tissues, especially in CD4^+^ T cells (**Supplementary Figure S6C**). To reveal the intrinsic structure and potential functional subsets of the overall T-cell populations, we performed unsupervised clustering of all T cells *via* t-SNE and UMAP algorithm and subsequently identified 12 distinct subsets of T cells, including 8 clusters of CD8^+^ cells and 4 clusters of CD4^+^ cells (**Figures 2A**, **3A** and **Supplementary Figures S7A, S8A**). The expression of signature genes and known functional markers indicated clusters of CD8^+^ cells (naïve, effector, proliferative, activated or exhausted) cells, conventional CD4^+^ (naïve, effector or activated) cells, CD4^+^ Tregs and a few clusters that were not well defined (**Figures 2B**, **3B** and **Supplementary Figures S7B, C, S8B, C**).

We observed that the overall number of CD8^+^ T cells in tumor tissues was lower than that in paracancer tissues (21), indicating that the infiltration efficiency of CD8^+^ T cells was low and might be associated with a poor prognosis (**Figure 2C**). We determined the cell number and proportion of each cellular subset (**Figure 2D**). The percentage of CD8-C1-*PDCD1* (CD8^+^ exhausted T cells) within CD8^+^ T cells isolated from tumor tissues and paracancer tissues was much higher than other cell types, revealing the potential enrichment of CD8^+^ exhausted T cells in the TME. GO enrichment analysis showed that CD8-C1-*PDCD1* (CD8^+^ exhausted T cells) showed a state of loss of function, persisted in the tumor tissues but responded poorly to the tumor cells (26, 27). These CD8^+^ exhausted T cells in the TME expressed high levels of *PDCD1* and could be rescued from the unresponsive and depleted state by ICB treatment (**Figure 2E**).

We applied the partition-based graph abstraction (PAGA) algorithm to order CD8^+^ T cells in pseudotime to indicate their developmental trajectories in SUPS (**Figure 2F**). We removed CD8-C6-*APOE*, *TUBA1B*, CD8-C7-*CCL5* and CD8-C8-*APOE* (clusters not well defined) due to their low cell numbers. Most cells from each cluster were gathered based on similar gene expression, and variant subsets formed a relative process in pseudotime. We observed CD8^+^ T-cell trajectories that began with CD8-C2-*LEF1* (CD8^+^ naïve T cells), followed by CD8-C3-*MKI67* (CD8^+^ proliferative T cells), CD8-C4-*GZMB* (CD8^+^ effector T cells) and CD8-C5-*IL7R* (CD8^+^ activated T cells), and ended with CD8-C1-*PDCD1* (CD8^+^ exhausted T cells). Those exhausted CD8^+^ T cells were highly enriched at the late period of pseudotime, demonstrating that the CD8^+^ T-cell state transformed from activation to exhaustion. The Monocle 2 and Monocle 3 algorithms confirmed the trajectories (**Supplementary Figure S7D**, **Figure 2G**). Using RNA velocity, a method inferring precursor progeny cell dynamics, we observed a clear directional flow from CD8^+^ proliferative T cells to CD8^+^ exhausted T cells. The proliferation in CD8^+^ T cells was most profound during the early stages of dysfunction (**Figure 2H**), which is consistent with a study of melanoma (28). We analyzed gene expression patterns involved in CD8^+^ T-cell-state transitions. The expression of genes related to “positive regulation of immune system process” decreased significantly along the pseudotime axis, while the expression of genes related to “cellular response to cytokine stimulus” increased significantly. The levels of genes related to the “positive regulation of cell adhesion” initially increased and then decreased along the pseudotime axis (**Figure 2I**). We identified 8 groups of DEGs along the trajectory of CD8-C1-*PDCD1* (CD8^+^ exhausted T cells). First, the naïve T-cell markers *CCR7*, *LEF1* and *TCF7* were reduced following the trajectory (29). The subsequent cell groups increased in abundance at the end of the trajectory and were characterized by effector *TNFSF9*, cytotoxicity *GZMK*, *GZMA*, *NKG7*, and early markers of general exhaustion *PDCD1*, *CTLA4* and *TIGIT*. In the last two groups, the early-activating genes *TGFB1*, *GZMM* and *TNF* increased midway through the trajectory but decreased thereafter. In each gene set, we authenticated several genes that were previously unidentified as T-cell markers (for example, *COTL1* and *PARK7* as differentiated exhausted CD8^+^ T-cell markers) (**Figure 2J**). Coactosin-like 1 (Cotl1) is another ADF-H protein that binds actin and was also shown to enhance biosynthesis of pro-inflammatory leukotrienes (LT) in granulocytes (30). Parkinson protein 7 (PARK7) has been found to play an inflammatory role in non-gestational tissues (31). The majority of the genes are related to regulator of inflammatory.

The percentage of CD4-C1-*CD69* (CD4^+^ activated T cells) among CD4^+^ T cells isolated from tumor tissues and paracancer tissues was much higher than other cell types, indicating the potential enrichment of CD4^+^ activated T cells in the TME (**Supplementary Figure S8D**). GO enrichment analysis showed that cluster CD4-C2-*FOXP3* (CD4^+^ Tregs) was enriched in genes related to functions involving “lymphocyte activation” and “response to cytokine” (**Figure 3C**) (32). Importantly, we also found that CD4-C2-*FOXP3* (CD4^+^ Tregs) and CD4-C3-*NKG7* (CD4^+^ effector T cells) positively expressed inhibitory receptors and ligands (IRs), including *TIGIT*, *CTLA4*, *PDCD1* and *LAG3*. IRs were associated with the exhaustion process of dysfunctional TIICs, suggesting that these cells became exhausted after initial activation. Tregs possessed relatively high levels of the immune inhibitory molecules *TIGIT*, *CTLA4*, *PDCD1* and *TNFRSF18*, which may contribute to Treg-mediated suppression of antitumor immune responses in the SUPS. Recently, anti-TIGIT therapeutics have drawn great attention in treating colorectal cancer, breast cancer and melanoma by modulating the activities of CD8^+^ T cells, Tregs and NK cells. We also noticed that *TIGIT* was widely expressed in CD8^+^ T cells, corresponding to a gradual loss of responsiveness (**Supplementary Figure S6D**). In the new era of immunotherapies, ICIs, including antibodies against PD-1 (nivolumab) and CTLA4 (ipilimumab), are widely used for cancer treatment. ICIs act by blocking the inhibitory receptors of the immune system on T cells (PD-1 and CTLA4) and thereby activate tumor-specific T cells to destroy tumor cells. Recently, nivolumab in combination with ipilimumab was reported to have survival benefits in patients suffering from hepatic melanoma of unknown primary origin. These messages illustrate that TIGIT, CTLA4 and PDCD1 blockade could be effective therapy for SUPS (26, 27).

Next, we analyzed CD4-C2-*FOXP3* (CD4^+^ Tregs) and obtained a list of 54 exhaustion-specific genes by comparing exhausted and non-exhausted CD4^+^ T cells. Multiple known exhaustion markers, such as *HAVCR2*, *PDCD1*, *ENTPD-1*, *CTLA4*, *TIGIT*, *TNFRSF9* and *CD27*, were selected. The 54-gene list also contained several little-described genes (21), such as *MYO7A*, *TOX* and *CXCL13*, as well as novel exhaustion markers, such as *LAYN*, *PHLDA1* and *SNAP47* (**Figure 3D**). Higher expression of the membrane fusion protein SNAP47 (synaptosome associated protein 47) was associated with poor prognosis in hepatocarcinoma (21). Pleckstrin homology-like domain, family A, member 1 (PHLDA1) has been reported to be a negative regulator of proinflammatory cytokine production (33). PHLDA1 plays an anti-inflammatory role through inhibiting the TLR4/MyD88 signaling pathway with the help of Tollip. Based on The Cancer Genome Atlas (TCGA) data, high levels of *LAYN* were associated with a poor prognosis (**Figures 3E, F**). *LAYN*, encoding layilin, was recently reported to be highly expressed in Tregs isolated from hepatocellular carcinoma. In addition, *LAYN* is linked to the suppressive function of tumor Tregs and exhausted CD8^+^ T cells. Thus, our data not only confirmed previously identified genes associated with exhausted CD4^+^ T cells and Tregs but also revealed additional markers for these cell types (34).

Similarly, using RNA velocity (29, 35), we observed a clear directional flow from CD4-C4-*TCF7* (CD4^+^ naïve T cells) to CD4-C2-*FOXP3* (CD4^+^ Tregs) (**Figure 3G**). The induced Tregs (iTregs) develop from peripheral naïve T cells under the induction of low-dose antigens or immunosuppressive cytokines. Tregs develop separately as iTregs and natural Tregs (nTregs) due to their different origins (36–38). Therefore, Tregs were not placed together with other CD4^+^ T cells for trajectory analysis. We applied the PAGA algorithm to order CD4^+^ T cells in pseudotime to indicate their developmental trajectories. Trajectories began with CD4-C4-*TCF7* (CD4^+^ naïve T cells), followed by CD4-C1-*CD69* (CD4^+^ activated T cells) and CD4-C3-*NKG7* (CD4^+^ effector T cells) (**Figure 3H**). The Monocle 3 algorithm confirmed the trajectories (**Figure 3I**), and the profiling of marker genes confirmed their functional annotation (**Supplementary Figure S8E**). We analyzed gene expression patterns involved in CD4^+^ T-cell-state transitions (39). The genes associated with “myeloid cell differentiation” and “immune system process” decreased significantly along the pseudotime axis, while the genes related to “T cell differentiation” and “T cell activation” increased markedly (**Supplementary Figure S8F**).

### Two distinct states of tumor-enriched macrophages

**Figure 4:**
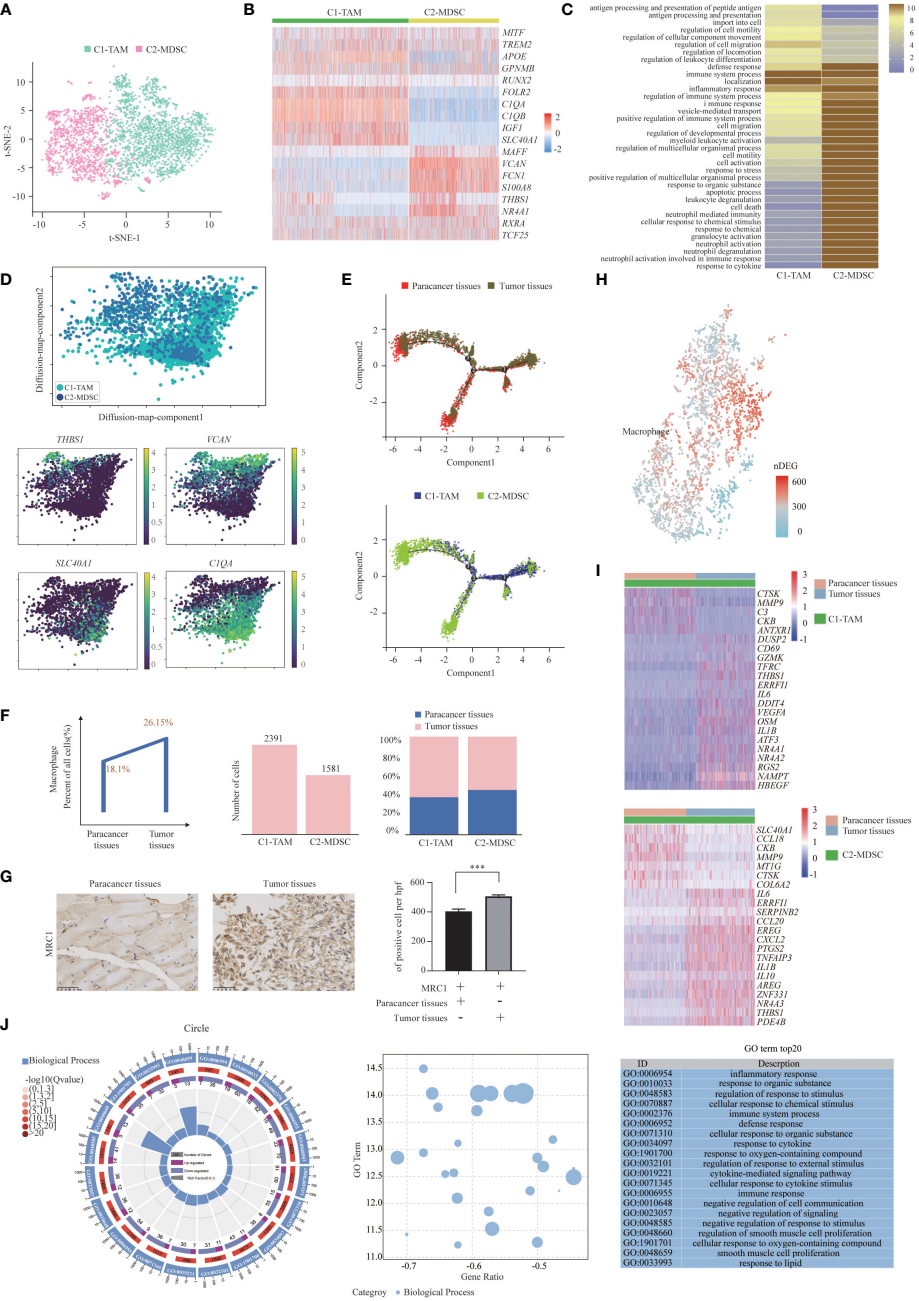
Two Distinct States of Tumor-Enriched Macrophages. **(A)** t-SNE plot showing the two main subsets of Macrophages. **(B)** Heatmap showing specific marker genes in each Macrophages cluster. **(C)** Functional enrichment analysis of upregulated genes in each Macrophages cluster was performed with GO analysis. **(D)** Diffusion map showing the continuous connection of the two macrophage states (upper) and signature gene expression based on 10x (lower). **(E)** The Monocle 2 trajectory plot showed the dynamics of TAMs and MDSCs. **(F)** The percentages of Macrophages in paracancer tissues and tumor tissues (left). The cell number (middle) and proportion (right) of each cluster. **(G)** Immunohistochemistry of SUPS showed the expression of MRC1 in paracancer tissues and tumor tissues. Quantification of IHC staining from tumor tissues and paracancer tissues (n = 3) displayed as the average number of positive cells per high-powered field (×200). Data are shown as the mean ± SEM. ***P <0.001. **(H)** Number of DEGs between paracancer tissues and tumor tissues within Macrophages projected onto the t-SNE map. Number of DEGs between paracancer tissues and tumor tissues. DEG: |log fold change| > 0.5, adjusted *P*< 0.05 determined by Wilcoxon rank-sum test. **(I)** DEGs of TAMs and MDSCs in tumor tissues vs paracancer tissues in SUPS were analyzed. **(J)** GO term analysis of DEGs in Macrophages of tumor tissues versus paracancer tissues was performed. The first lap indicates the top 20 GO terms and the number of the genes corresponds to the outer lap. The second lap indicates the number of genes in the genome background and Q values for enrichment of the upregulated genes for the specified biological process. The third lap indicates the ratio of the upregulated genes (deep purple) and downregulated genes (light purple). The fourth lap indicates the enrichment factor of each GO term. GO, gene ontology.

We detected a total of 3972 macrophages that formed 2 clusters (**Figure 4A**). Although genes upregulated in the C2-MDSC cluster were enriched for signatures of myeloid-derived suppressor cells (MDSCs), those in the C1-TAM cluster simultaneously resembled the signatures of tumor-associated macrophages (TAMs) and M1 and M2 macrophages (**Figure 4B**). The coexistence of M1 and M2 signatures indicated that TAMs are more complex than the classical M1/M2 model, which was consistent with a previous study. Specifically, MDSC-like macrophages highly expressed the *S100A* family genes *FCN1* and *VCAN*, whereas they expressed low levels of HLA-related genes (40). In contrast, TAM-like macrophages expressed a set of genes (*APOE*, *C1QA*, *C1QB* and *TREM2*), which was found previously to be expressed in the TAMs of lung cancer. Furthermore, two additional genes, *SLC40A1*, which encodes ferroproteins, and *GPNMB*, which encodes type I transmembrane glycoprotein, showed high levels in TAM-like macrophages. The transcription factors of these two clusters were diverse; that is, TAM-like macrophages preferentially expressed *MITF*, *RUNX2* and *MAF*, and MDSC-like macrophages expressed high levels of *NR4A1*, *RXRA* and *TCF25* (**Figure 4B**). Enrichment analysis of upregulated gene subsets showed that the function of TAMs was mainly “regulation of cell migration” and “regulation of cell motility”. MDSCs primarily function in “inflammatory response” and “regulation of immune system process” (**Figure 4C**).

A diffusion map of their global transcriptomes showed that the C2-MDSC cluster (MDSC-like macrophages) and the C1-TAM cluster (TAM-like macrophages) formed a continuum but with distinct expression features (**Figure 4D**). The t-SNE analysis is instrumental in revealing the heterogeneity of distinct macrophage clusters. However, the clusters may share common differentiation trajectories. Most macrophages were arranged into a major trajectory with two minor bifurcations by pseudotime ordering. Macrophages from different samples are widely distributed in the pseudotime space. Macrophages in paracancer tissues occupied the lower part, while macrophages in the tumor tissues were located in the higher position, indicating that the cells in the lower part may be the origin of differentiated macrophages. MDSCs were mainly distributed in the left lower branch, while TAMs occupied the right upper part, which also suggests that the cells in the lower part may be the starting point of the differentiation of macrophages (**Figure 4E**). We speculate that macrophages tend to transform the MDSC phenotype to the TAM phenotype (41).

The numbers of TAMs and MDSCs isolated from tumor tissues were higher than those isolated from paracancer tissues, demonstrating that TAMs and MDSCs are preferentially recruited to the TME (**Figure 4F**) (42). Indeed, the number of macrophages was increased in tumor tissues compared with the corresponding paracancer tissues (**Figure 4G**). The slight increase in macrophages in tumor tissue indicates that macrophage immunotherapy may be effective in patients with SUPS (41). After defining the macrophages in our dataset, we identified the DEGs in tumor tissues and paracancer tissues of SUPS. We authenticated the differential gene set for these macrophages, allowing for a more in-depth analysis of regulatory pathways (**Figure 4H**). We focused on the genes with twofold upregulation or downregulation in macrophages in tumor tissues compared with paracancer tissues. A heatmap profiles showed that *CTSK*, *MMP9*, *CKB*, *CCL18* and *COL6A2* were upregulated in macrophages in tumor tissues (**Figure 4I**). GO enrichment analysis demonstrated that the majority of the downregulated genes in the macrophage subsets were related to “inflammatory response” and “regulation of response to external stimulus” (**Figure 4J**).

### Two distinct states of tumor-enriched osteoclasts

**Figure 5:**
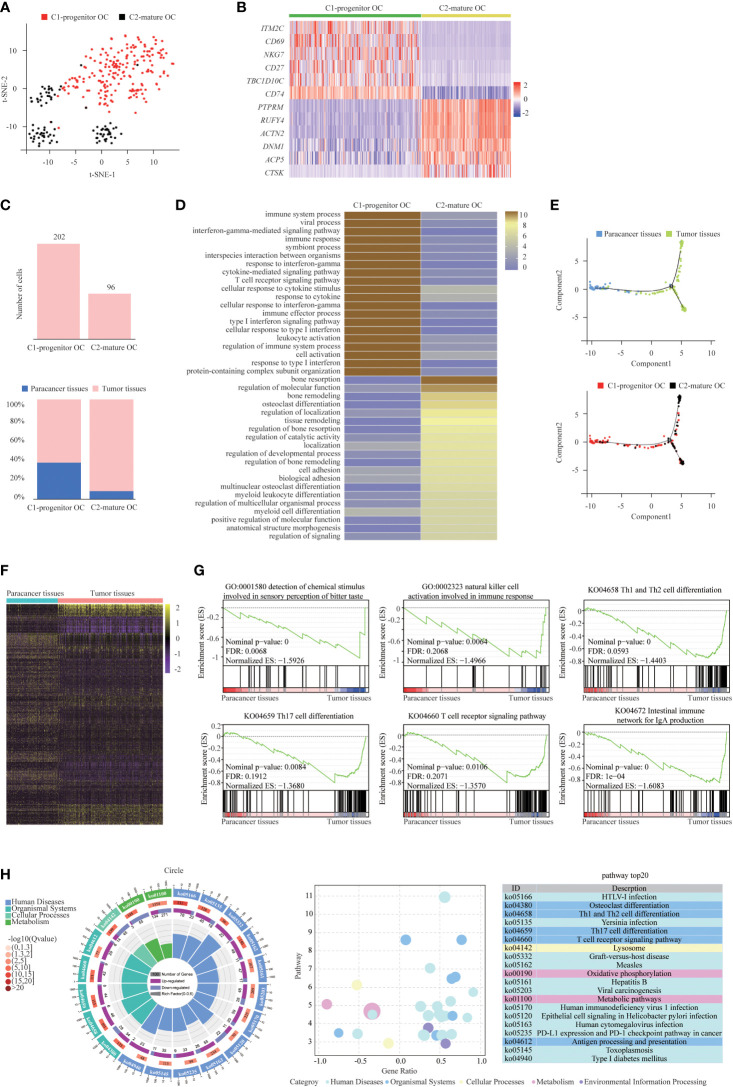
Two Distinct States of Tumor-Enriched Osteoclasts. **(A)** t-SNE plot showing the two main subsets of Osteoclasts. **(B)** Heatmap showing specific marker genes in each Osteoclasts cluster. **(C)** The cell number and proportion of each Osteoclasts cluster. **(D)** Functional enrichment analysis of upregulated genes in each Osteoclasts cluster was performed with KEGG analysis. **(E)** The Monocle 2 trajectory plot showing the dynamics of C1-progenitor OC and C2-mature OC. **(F)** DEGs of Osteoclasts in tumor tissues vs paracancer tissues in SUPS was analyzed. **(G)** GSEA analysis was performed on Osteoclasts of tumor tissue vs paracancer tissue. **(H)** KEGG analysis of DEGs in Osteoclasts of tumor tissues versus paracancer tissues was performed. The first lap indicates the top 20 KEGG terms and the number of the genes corresponds to the outer lap. The second lap indicates the number of genes in the pathway and Q values for enrichment of the upregulated genes for the specified pathway. The third lap indicates the ratio of the upregulated genes (deep purple) and downregulated genes (light purple). The fourth lap indicates the enrichment factor of each KEGG pathway. KEGG, Kyoto Encyclopedia of Genes and Genomes.

Osteoclasts play a vital role in osteolysis and tumor growth in tumor tissues. Based on the t-SNE algorithm, two individual subsets of osteoclasts were identified with distinct levels of myeloid markers, such as *CD74*, and/or mature osteoclastic markers (such as *CTSK* and *ACP5*) (39) (**Figure 5A**). The subset described as C1-progenitor OC had high levels of the myeloid markers *CD74* and *CD27* and low levels of the OC markers *CTSK* and *ACP5*. The C2-mature OC subset expressed high levels of *CTSK* and *ACP5* and low levels of *CD74* (**Figure 5B**). Both progenitor osteoclasts and mature osteoclasts were more abundant in tumor tissues than in paracancer tissues, suggesting that progenitor osteoclasts and mature osteoclasts may be enriched in the TME (**Figure 5C**). Enrichment analysis showed that the main functions of the C1-progenitor OC subset were “type I interferon signaling pathway” and “response to cytokine”. The main functions of the C2-mature OC subset were “bone resorption” and “regulation of bone resorption” (**Figure 5D**).

**Figure 6:**
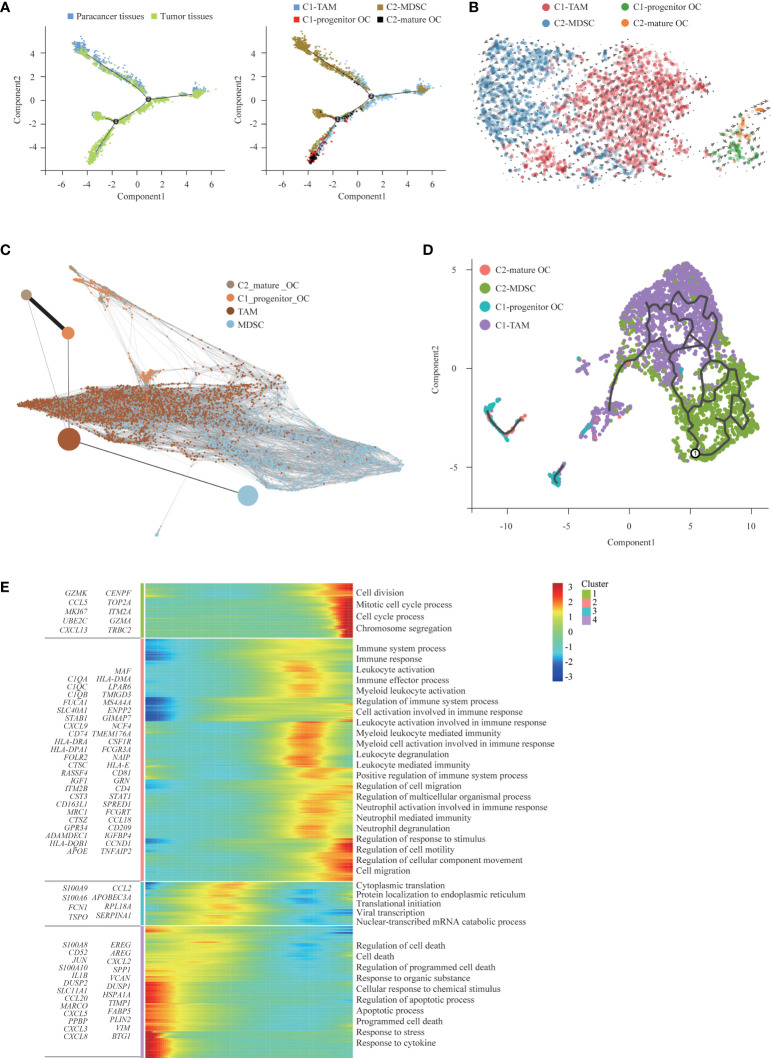
The trajectory analysis of osteoclasts and macrophages. **(A)** Monocle 2 analysis, **(B)** RNA velocity analysis, **(C)** PAGA analysis, and **(D)** Monocle 3 analysis on Osteoclasts and Macrophages phenotypes independently confirming the maturation trajectories. **(E)** The DEGs in Osteoclasts and Macrophages along the pseudotime were hierarchically clustered into different subsets. The top annotated GO terms in each cluster were provided.

Osteoclasts are specialized cells derived from the monocyte/macrophage hematopoietic lineage. They develop and adhere to the bone matrix and then secrete acid and lytic enzymes to degrade the bone matrix in a specialized extracellular compartment. Increased bone resorption is the result of osteoclast formation induced by tumor cells, and osteoclast formation facilitates bone resorption. Bone is a heterogeneous environment that is beneficial for the growth of tumor cells. Among the different cell types presented in bone, osteoclasts are crucial players in the so-called “vicious cycle”. This phenomenon is triggered by tumor cells. Eventually tumor proliferation and bone deregulation occur, which promote the development of bone metastasis (43). Subsequently, we performed trajectory analysis of osteoclasts to infer the osteoclast maturation process in SUPS. The mature osteoclasts were highly enriched at the late period of pseudotime, demonstrating that the osteoclast state transformed from progenitor to maturation (**Figure 5E**). Then, we analyzed the trajectory of macrophages and osteoclasts. MDSCs mainly occupied the left upper branch, while TAMs were primarily located in the right lower part. Both clusters of osteoclasts were concentrated at the end of the lower branch, suggesting that macrophages tended to differentiate into osteoclasts (**Figure 6A**) (44, 45). RNA velocity analysis and the Monocle 3 and PAGA algorithms confirmed these trajectories (**Figures 6B-D**). We analyzed the gene expression patterns involved in osteoclast and macrophage differentiation. Genes related to “regulation of programmed cell death” decreased observably along the quasi-time axis. Genes associated with “mitotic cell cycle process” increased dramatically along the pseudotime axis. Genes regarding “myeloid leukocyte mediated immunity” initially increased and subsequently decreased along the quasi-time axis (**Figure 6E**).

After defining the osteoclasts in our dataset, we identified the DEGs in tumor tissues and paracancer tissues. We identified the differential gene set for these osteoclasts that allowed a more in-depth analysis of regulatory pathways (**Figure 5F**). Gene set enrichment analysis (GSEA) demonstrated that osteoclasts in SUPS negatively regulated the humoral immune response (**Figure 5G**). KEGG enrichment analysis showed that most of the upregulated genes of osteoclasts in SUPS were related to “PD-L1 expression and PD-1 checkpoint pathway in cancer” and “T cell receptor signaling pathway” (**Figure 5H**).

### Gene expression heterogeneity in Fibroblast subsets was identified in the SUPS patient

**Figure 7:**
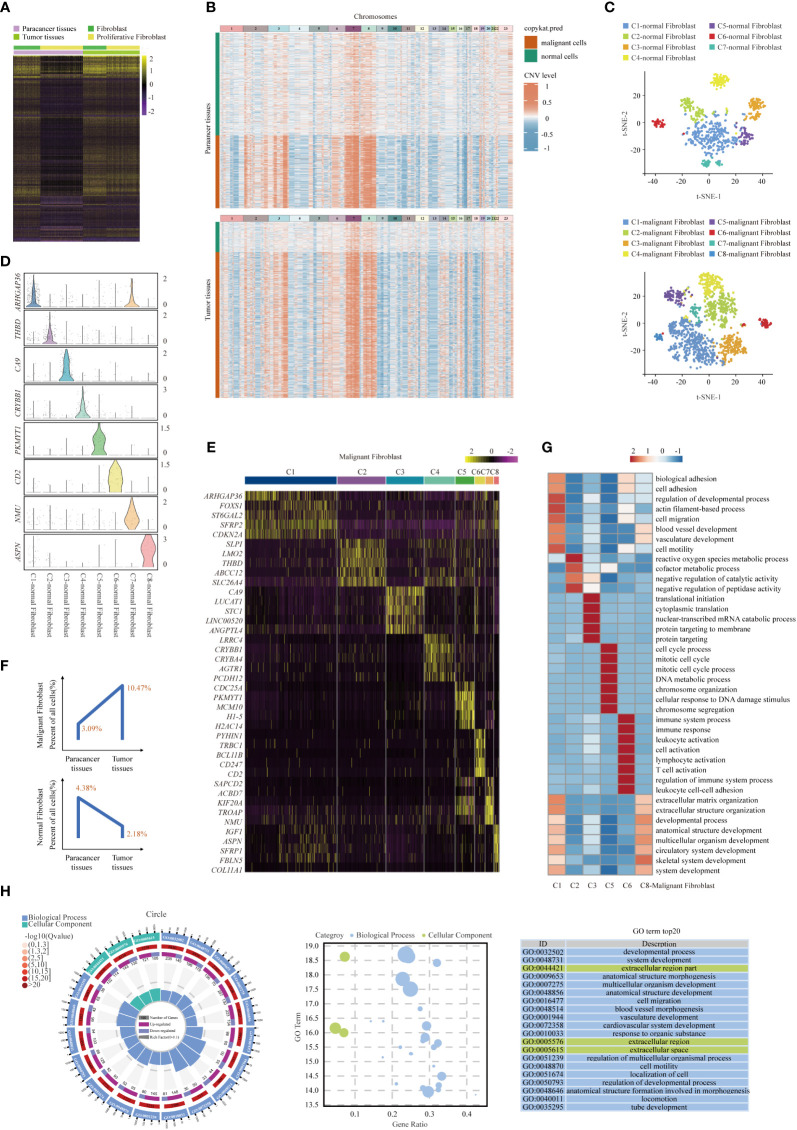
Gene expression heterogeneity of fibroblast subsets was identified in the SUPS. **(A)** DEGs of fibroblasts in tumor tissues vs paracancer tissues in SUPS were analyzed. **(B)** Clustered heat map of 2395 scRNA-seq copy number profiles estimated by CopyKAT. Fibroblasts were identified by CopyKAT and divided into malignant fibroblasts and normal fibroblasts. **(C)** t-SNE plot showing the seven main subsets of normal fibroblasts and the eight main subsets of malignant fibroblasts. **(D, E)** Violin plot and Heatmap showing specific marker genes in each malignant fibroblasts cluster. **(F)** The percentages of malignant/normal fibroblasts in paracancer tissues and tumor tissues. **(G)** Functional enrichment analysis of upregulated genes in each malignant fibroblasts cluster was performed with GO analysis. **(H)** GO term analysis of DEGs in malignant fibroblasts of tumor tissues versus paracancer tissues was performed.

As an important cell component in the disease lesion, Fibroblasts within paracancer tissues and tumor tissues were compared, and various DEGs were identified (**Figure 7A**). We found abundant DEGs in tumor and paracancer tissues, so we inferred that cancerous fibroblasts exist in fibroblasts (25). Copy number karyotyping of aneuploid tumors (CopyKAT) (46) can infer cell chromosome multiples by analyzing single-cell transcriptome data and then infer whether normal cells (diploid) or malignant cells (aneuploid). CopyKAT can further cluster tumor cells and identify different subsets. CopyKAT does not need normal cells as a reference and can automatically find diploid cells as normal cells, making up for the shortcomings of inferCNV and HoneyBadger. Therefore, fibroblasts were identified by CopyKAT and divided into malignant fibroblast and normal fibroblast groups (**Figure 7B**). To determine the intrinsic structure and potential functional subtypes of the entire fibroblast population, we performed unsupervised clustering of these two types of cells to examine their heterogeneity (**Figure 7C**). According to the DEGs, normal fibroblasts were divided into 7 clusters (**Supplementary Figure S9A**): C1-normal Fibroblast, C2-normal Fibroblast, C3-normal Fibroblast, C4-normal Fibroblast, C5-normal Fibroblast, C6-normal Fibroblast and C7-normal Fibroblast. Tumor-associated fibroblasts were divided into 8 clusters (**Figures 7D, E**): C1-malignant Fibroblast, C2-malignant Fibroblast, C3-malignant Fibroblast, C4-malignant Fibroblast, C5-malignant Fibroblast, C6-malignant Fibroblast, C7-malignant Fibroblast and C8-malignant Fibroblast (22, 25, 47, 48). Besides, we found that normal fibroblasts are mainly distributed in paracancer tissues, while malignant fibroblasts are primarily distributed in tumor tissues (**Figure 7F** and **Supplementary Figures S9B, C**).

Enrichment analysis of upregulated gene subsets showed that the primary functions of C1-malignant Fibroblasts were “blood vessel development” and “cell migration”. The main functions of C6-malignant Fibroblasts were “immune system process”, “leukocyte activation” and “T-cell activation” (**Figure 7G**). In addition, GO enrichment analyses revealed that within the malignant cells of SUPS, fibroblasts were enriched for genes associated with “cell activation” and “inflammatory response” (**Figure 7H**). Malignant fibroblasts (for example, C1-malignant Fibroblasts and C6-malignant Fibroblasts) in the TME play crucial roles in tumor growth, angiogenesis, metastasis and immune response. Thus, targeting malignant fibroblasts could represent a potential strategy for treating this patient (48).

### Cell communication networks in SUPS

**Figure 8:**
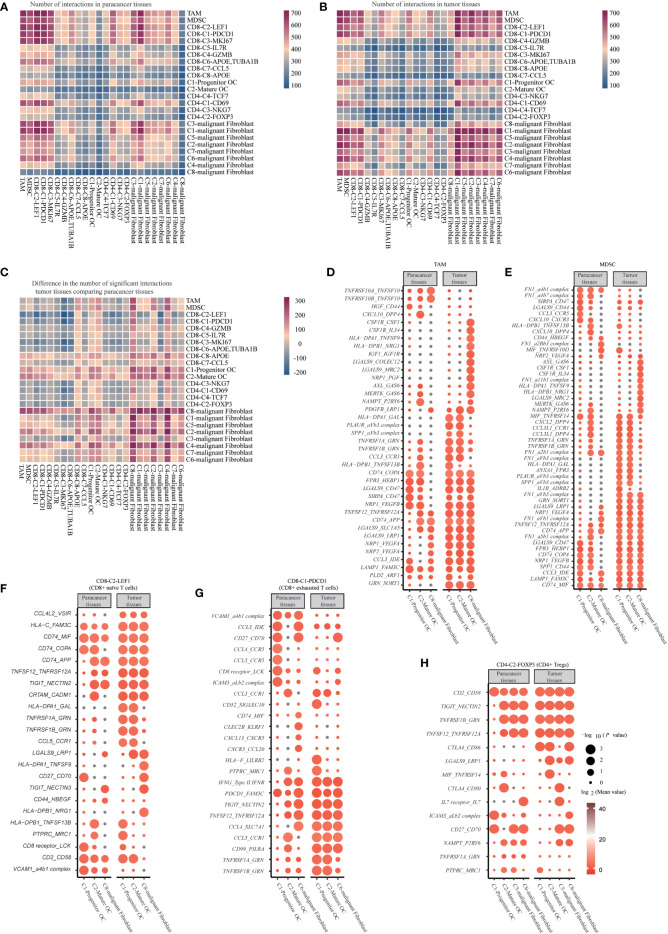
Cell Communication Networks in SUPS. **(A, B)** Heatmap of the number of ligand-receptor pairs each cell has (each row/column represents a cell, and the number of ligand-receptor pairs each cell has is colored, the bluer the cell has fewer ligand-receptor pairs, and the redder the cell has more ligand-receptor pairs). **(C)** Results of the ratio of the number of ligand-receptor pairs in tumor tissue compared with paracancer tissue. **(D-H)** Overview of selected ligand-receptor interactions of Macrophages, Osteoclast, Malignant fibroblast cells, and T cells. P values are indicated by circle size, Mean values are indicated by circle color.

We used CellphoneDB to predict receptor-ligand interactions. First, we calculated the interactions in the cell types from tumor tissues and paracancer tissues separately. We observed that cells from tumor tissues had more potential for interaction than those from paracancer tissues, especially in malignant fibroblasts, osteoclasts, macrophages and several kinds of T cells (29). Interestingly, we found a cellular communication network between tumor cells and immune cells mediated by immune checkpoint ligand-receptor interactions (**Figures 8A-C**).

The pro-apoptotic interaction between CD8-C2-*LEF1* (CD8^+^ naïve T cells) and mature osteoclasts, progenitor osteoclasts or C8-malignant Fibroblasts (*TNFSF12_TNFRSF12A*, *TNFRSF1A_GRN* and *TNFRSF1B_GRN*) was increased. Mature osteoclasts, progenitor osteoclasts or C8-malignant Fibroblasts express *NECTIN2* and *NECTIN3*, thereby transmitting inhibitory signals to *TIGIT* on CD8-C2-*LEF1* (CD8^+^ naïve T cells). Some new communication links have been observed between mature osteoclasts, progenitor osteoclasts, C8-malignant Fibroblasts and CD8^+^ T cells in tumor tissues, indicating that T cells are recruited to tumor tissues (*CCL4L2_VSIR* and *CCL5_CCR1*). CD8-C2-*LEF1* (CD8^+^ naïve T cells) showed inhibitory interactions with mature osteoclasts or progenitor osteoclasts (*HLA-DPA1_GAL* and *HLA-FAM3c*), or with C8-malignant Fibroblasts (*HLA-DPB1_NRG1*). The proapoptotic interactions of CD8-C1-*PDCD1* (CD8^+^ exhausted T cells) with mature osteoclasts, progenitor osteoclasts or C8-malignant Fibroblasts were increased (*TNFSF12_TNFRSF12A*, *TNFRSF1B_GRN* and *TNFRSF1A_GRN*), but T-cell-homing communications were weakened (*CCL4_CCR5*, *CCL5_CCR5* and *CXCR3_CCL20*). Mature osteoclasts, progenitor osteoclasts and C6-malignant Fibroblasts express *NECTIN2* and *FAM3C*, which transmit inhibitory signals to *TIGIT* and *PDCD1* on CD8-C1-*PDCD1* (CD8^+^ exhausted T cells), respectively. The costimulatory interaction between CD4-C2-*FOXP3* (CD4^+^ Tregs) and mature osteoclasts or progenitor osteoclasts (*TNFRSF1B_GRN*, *TNFSF12_TNFRSF12A* and *MIF_TNFRSF14*) increased. *NECTIN2*, *CD80* and *CD86* expressed on mature osteoclasts, progenitor osteoclasts, C5-malignant Fibroblasts or C6-malignant Fibroblasts, transferred the suppressive signals to *TIGIT* and *CTLA4* on CD4-C2-*FOXP3* (CD4^+^ Tregs), respectively. It is noteworthy that CD4-C2-*FOXP3* (CD4^+^ Tregs) possessed relatively high levels of adhesion molecules, including *CD2* and *ICAM3*. The corresponding receptors, including *CD58* and the *aLb2* complex, are widely expressed by mature osteoclasts, progenitor osteoclasts, C5-malignant Fibroblasts and C6-malignant Fibroblasts, which can enhance the adhesion and growth of SUPS. Some new interactions have been observed between MDSCs and mature osteoclasts or progenitor osteoclasts in tumor tissues, suggesting that MDSCs are recruited to tumor tissues (*CCL3L1_CCR1*, *CXCL2_DPP4* and *CCL3L1_DPP4*). Furthermore, angiogenic signals increased between MDSCs and mature osteoclasts, progenitor osteoclasts or C8-malignant Fibroblasts (*NRP2_VEGFA*, *NRP1_VEGFA* and *NRP1_VEGFB*). In addition, angiogenic signals (*NRP1_VEGFB*, *IGF1_IGF1R*, *NRP1_VEGFA* and *NRP2_VEGFA*) and costimulatory effects (*TNFRSF1A_GRN* and *TNFRSF1B_GRN*) increased between TAMs and mature osteoclasts, progenitor osteoclasts or C8-malignant fibroblasts (**Figures 8D–H**) (11, 25, 49). Overall, these data depict an interactive immune environment in SUPS.

## Discussion

Here, we provide a comprehensive analysis of scRNA-seq data generated from SUPS. The deep transcriptome for 18433 individual cells provided an extensive resource for understanding the multidimensional characterization of SUPS, especially in the tumor immune microenvironment. The higher resolution provided by our dataset was exemplified by the identification of 19 large subsets as well as unique subpopulations, such as CD8^+^ exhausted T cells and CD4^+^ Tregs (50). The high quantity and quality of single-cell data allowed us to map their developmental trajectory. In addition, pseudotime analysis and RNA velocity analysis permits us not only to confirm their differentiated relationships in various clusters but also to deduce their activation status in the cancer microenvironment.

The infiltration of T cells and their characteristics are usually associated with prognostic outcomes. In our study, we determined that *PDCD1*, *CTLA4* and *TIGIT* are involved in the exhaustion of CD8^+^ and CD4^+^ T cells in SUPS. Their receptors, such as *FAM3C*, *CD80/CD86* and *NECTIN2*, are widely expressed in malignant fibroblasts, which may be the cause of poor prognosis (27). Antibody blockade of the PD-1 pathway has been shown to reinvigorate exhausted CD8^+^ T cells with *PDCD1* expression. We discovered that *LAYN* was highly expressed in CD4^+^ Tregs, which was speculated to be associated with CD4^+^ Treg depletion and a poor prognosis. A previous study revealed a regulatory role of *LAYN* in Treg function. Similarly, some studies have proven the induction of *LAYN* after the activation of exhausted CD8^+^ T cells and CD4^+^ Tregs (34). Furthermore, the overexpression of *LAYN* on CD8^+^ T cells in human blood leads to a significant reduction in the production of IFN-g, which is a key cytokine involved in the tumor-killing activity, and supports *LAYN* as a negative regulator. TCGA data showed that high *LAYN* levels were associated with short survival time of various cancers. More studies are needed to further investigate the function of *LAYN* and other genes related to depleted CD4^+^ Tregs in SUPS. We identified CD4^+^ effector T cells and CD4^+^ activated T cells, which shared similar gene expression characteristics to CD8^+^ effector T cells and CD8^+^ activated T cells, implicating their cytotoxic functions. CD4^+^ effector T cells also highly expressed *CCL5* and *GZMA*, which appear to be in a mixed state among known subtypes of T helper cells but are more similar to effector cells due to the expression of cytotoxic molecules (*GZMK*, *NKG7* and *GNLY*). Therefore, increasing the similarity between these cells and cytolytic CD4^+^ T cells might be a new strategy for SUPS immunotherapy (51).

SUPS is an aggressive form of soft tissue sarcoma. SUPS is extremely rare, with limited information on its pathogenesis, clinical and radiological features, pathological findings and therapeutic outcomes. The pathophysiology remains elusive, and treatment options are limited (5). Advances in scRNA-seq technology have enabled a comprehensive analysis of the immune system in an unbiased way at the single-cell level. To our knowledge, this study is the first to analyze SUPS using scRNA-seq technology (16). Analysis of our single-cell database revealed the detailed characteristics of SUPS-infiltrating cells in the microenvironment, including their aggregation, dynamics and developmental trajectory, as well as unique characteristics in tumor tissues and the corresponding paracancer tissues. Single cell transcriptomics has revealed intratumoral heterogeneity within many cancer types, identifying cell populations that drive drug resistance, predict metastatic risk and mediate plasticity (52, 53). However, studies often suffer from a lack of normal tissue comparisons that can be used to identify tumor-specific biology, and loss of spatial information after tissue dissociation. Recent advances, however, enable simultaneous capture of the locations of dozens of cell types within the TME, which is critical for understanding SUPS (54). Orthogonal integration of single-cell and high-dimensional spatial data from both normal and diseased tissues should therefore facilitate the dissection of TME cellular communication.

In conclusion, we reported the first case of SUPS identified by scRNA-seq, described the characteristics of the TME, identified markers and particular clusters related to cancer immunotherapy, and provided a therapeutic basis. We found that malignant fibroblasts and osteoclasts are significantly enriched in tumor tissues, and most of them are actively undergoing EMT, which leads to cancer invasion and metastasis. The transition from TAMs to osteoclasts also promotes tumor invasion and metastasis. We detected that high levels of CD8^+^ exhausted T cells accumulate in SUPS with abundant aggregation during the late period of pseudotime. In our study, the upregulation of *PDCD1*, *CTLA4*, *TIGIT* and *LAYN* suggests that inhibitors of these biomarkers may be effective against SUPS. At present, there are no guidelines for the use of ICIs in SUPS. We confirmed that PD-1 ICIs can be used as first-line treatment for patients with SUPS.

## Experimental section

### Human studies statement

The present study has been reviewed and approved by Sir Run Run Hospital Nanjing Medical University.

### Sample preparation and scRNA-seq

Fresh lesions were stored in tissue preservation solution and processed on ice after the surgery within 30 mins. Single-cell suspensions of the collected tissues were prepared through mechanical dissociation and enzymatic digestion within 16 h after surgery. Briefly, tissues were cut into pieces that were 2-4 mm in size and transferred to a tube containing the enzyme mix. The tissues were incubated in an enzyme solution (collagenase, DNase I, and Dispase II; prepared in DMEM) at 37°C for 1 h. The tissue pieces were remixed by gentle pipetting at 20 min intervals during incubation. Each cell suspension was transferred to a new 50 ml (15 ml tube for biopsy samples) tube after being passed through a 70 µm strainer. The volume in the tube was readjusted to 50 ml (or 15 ml) with DMEM medium, and the contents were centrifuged to remove the enzymes. The supernatant was aspirated, the cell pellet was resuspended in 4 ml of DMEM medium, and the dead cells were removed using Ficoll-Paque Plus (GE Healthcare, Chicago, IL, USA) separation. Single cells were encapsulated in droplets using 10 × Genomics GemCode Technology and processed according to the manufacturer’s instructions. We prepared single-cell RNA-seq libraries with Chromium Single Cell 3' Gel Beads-in-emulsion (GEM) Library & Gel Bead Kit v3 according to the user manual supplied by the kit.

### Quality control metrics and data processing

Using CellRanger (version 3.0.0), reads were mapped to the reference genome and annotated as specific genes. After the UMI was corrected and counted, the unfiltered feature-barcode matrix was obtained. According to the unfiltered feature-barcode matrix, CellRanger identifies and distinguishes cells and noncells in the data and draws a rank-plot to intuitively reflect the effective cell identification results. CellRanger filters cells automatically according to gene expression levels, and some abnormal cells will remain, so it is necessary to further filter abnormal cells before subgroup classification. Gel beads-in-emulsion (GEMs) containing multiple cells in each sample was first detected. DoubletFinder (https://github.com/ddiez/DoubletFinder) was used to calculate the probability of GEM multicellular (pANN value), and then the multicellular rate of each sample was calculated based on the relationship between the effective cell number given by 10 × (after CellRanger filtering) and the multicellular rate. The multicellular filtering threshold of each sample was determined, and multicellular filtering was carried out in turn. The number of expressed genes in a single cell or the same type of cell is generally maintained within a certain range (340.0-6800.0). If the value is too high, it may be that multiple cell types are wrapped in a GEM, so the barcode is eliminated. The total number of mRNAs that can exist in a single cell is limited (UMI <44000.0). If the total number of UMIs is too high, two or more cells may be contained in the same GEM, thus eliminating such cells. Mitochondrial gene expression is usually high among cells with an apoptosis rate >25.0%. High expression of mitochondrial genes indicates that the cells are in poor health induced by damage during the experiment, so these cells are excluded from subsequent analysis. After applying quality control metrics, single cells were included in downstream analyses. Library size normalization was performed with NormalizeData function in Seurat (version 3.1.1) to obtain the normalized count. Specifically, the global-scaling normalization method “LogNormalize” normalized the gene expression measurements for each cell by the total expression, multiplied by a scaling factor (10,000 by default), and the results were logtransformed. The most variable genes were selected using FindVariableGenes function (mean.function = FastExpMean, dispersion.function = FastLogVMR) in Seurat. Principal component analysis (PCA) was executed to reduce the dimensionality with RunPCA function in Seurat. Graph-based clustering was performed to cluster cells according to their gene expression profile using the FindClusters function in Seurat. Cells were visualized using a 2-dimensional t-SNE and UMAP algorithm in Seurat. We used the FindAllMarkers function (test.use = presto) in Seurat to identify marker genes of each cluster. For a given cluster, FindAllMarkers identified the positive markers compared with all other cells.

### Histological analysis

Human tissue specimens were provided by SIR RUN RUN HOSPITAL NANJING MEDICAL UNIWERSITY under an approved Institutional Review Board protocol. The specimens were collected within 30 min after the tumor resection and fixed in formalin for 48 hr. Dehydration and embedding in paraffin were performed following routine methods. For histopathological analysis, H&E staining was performed on formaldehyde-fixed, paraffin-embedded tissue samples. Microscopic analysis of the staining was evaluated by examining 3 sections from each tissue. The sections were observed using a laser scanning confocal microscope.

### Immunohistochemistry

Tissue sectioning and immunohistochemistry staining of formalin-fixed, paraffin-embedded SUPS specimens were performed. All sections were deparaffinized, rehydrated, and washed. Endogenous peroxidase was blocked using 3% hydrogen peroxide for 10 min. After water-bath heating for antigen retrieval, slides were incubated with primary antibodies followed by horseradish peroxidase (HRP)-linked secondary antibodies and diaminobenzidine staining (G1213-100UL, G1214-100UL). Hospital Pathology Department blinded to clinical data independently assessed staining results for SMA (unnecessary dilution, ZM-0003,ZSGB-BIO), CD68 (unnecessary dilution, ZM-0464, ZSGB-BIO), Ki67 (1:100, ZM-0166, ZSGB-BIO), CD34 (unnecessary dilution, ZM-0046, ZSGB-BIO), Desmin (unnecessary dilution, ZA-0686, ZSGB-BIO), EMA (unnecessary dilution, ZM-0095, ZSGB-BIO), S-100 (unnecessary dilution, ZM-0224, ZSGB-BIO), BCL-2 (unnecessary dilution, ZA-0536, ZSGB-BIO). Servicebio blinded to clinical data independently assessed staining results for CD3 (1:250, GB11014, Servicebio), CD4 (1:400, GB11064-1, Servicebio), CD8 (1:200, GB11068-1, Servicebio). Quantification was performed by counting positive cells in 6 to 10 high-powered fields (magnification, ×40) in a blinded fashion.

### Analysis of differentially expressed genes

DEGs were identified using the FindMarkers function (test.use = presto) in Seurat. We identified the DEGs based on the following criteria: |logFC| ≥ 0.25 and p_value_adj ≤ 0.05, and the percentage of cells where the gene is detected in the specific cluster is more than 25%. The identification of differentially upregulated genes was performed using the FindAllMarkers function (test.use = presto) in Seurat. The identified genes were differentially upregulated in each cell classification compared with other cell populations. We used the gene ontology (GO) and Kyoto Encyclopedia of Genes and Genomes (KEGG) pathway enrichment analysis to discover certain biological functions and pathways. GO and KEGG pathway enrichment analysis of DEGs were respectively performed using R based on the hypergeometric distribution.

### Pseudotime analysis

Trajectory analysis of CD8^+^ T cells, CD4^+^ T cells, macrophages and Osteoclasts were performed, respectively using Monocle and PAGA. The PAGA (35) (Partition based graph abstraction) (https://github.com/theislab/paga) graph was made using the preprocessed Seurat object. PAGA achieves consistent and topology-preserving embeddings by initializing an embedding of a fine-grained graph using the coordinates of a coarse-grained graph. After assigning the starting cell, the software automatically calculated the pseudo-time value of each cell by referring to the DPT algorithm. Single cell trajectory was analyzed using the matrix of cells and gene expressions by Monocle (http://cole-trapnell-lab.github.io/monocle-release/; https://cole-trapnell-lab.github.io/monocle3/docs/installation/). Monocle reduced the space down to one with two dimensions and ordered the cells (sigma = 0.001, lambda = NULL, param.gamma = 10, tol = 0.001). Once the cells were ordered, we could visualize the trajectory in the reduced dimensional space. The trajectory has a tree-like structure, including tips and branches.

### RNA velocity-based cell fate tracing

To perform the RNA velocity analysis, the spliced reads and unspliced reads were recounted by the velocyto python package based on previous aligned bam files of scRNA-seq data. The calculation of RNA velocity values for each gene in each cell and embedding RNA velocity vector to low-dimension space was done by following the scvelo python pipeline. We calculated the velocity-based cell transition matrix by transition_matrix () function from scvelo (https://scvelo.readthedocs.io/), of which the element was the Pearson correlation coefficient between the velocity vector and cell state difference vectors of the column cell as previously described. We estimated the destination of a cell by identifying the highest correlation value. Then Fisher’s exact test was performed on 2×2 cluster-by-cluster or cluster-by-tissue contingency tables to test the fate destinations of interested cell clusters. To infer the migration directions of T cell, osteoclast and macrophage, we first constructed partition-based graph abstraction for T cell population, osteoclast population, and macrophage population respectively, and then oriented edges among cell populations using RNA velocity information as previously described.

### Copy number karyotyping of aneuploid tumors

To distinguish malignant cells from normal cells in fibroblasts, we chose CopyKAT (copynumber karyotyping of tumors) software to calculate the CNV level of each cell (46). CopyKAT is a software that combines the integrative Bayesian method and hierarchical clustering to classify cells according to copy number. We use scRNAseq technology to get the gene expression data of fibroblasts in paracancer tissues and tumor tissues. The gene expression matrix of the unique molecular identifier (UMI) of two fibroblasts from different sources is the input of CopyKAT. The software cluster the UMI data after processing, and select the diploid cells with high confidence first. Then, using hierarchical clustering, tumor cells with significant differences from normal cells are obtained. For non-significant genomes, the Gaussian mixture model (GMM) is used to identify them one by one. Finally, get the gene expression profiles of malignant cells and normal cells.

### Cell-to-cell communication of scRNA-seq data

The ligand-receptor interactions among immune cells from the SUPS were mapped using the CellPhoneDB (55) algorithm (https://github.com/Teichlab/cellphonedb). Briefly, the algorithm allows the detection of ligand-receptor interactions between cell types in scRNA-seq data using the statistical framework described in refs. We took the union of the significant interactions found in tumor tissues and paracancer tissues to explore specific interactions. Next, we assessed the number of interactions that are shared and specific for tumor tissues and paracancer tissues and explored specific interactions indicated as curated (that is, annotated by the CellPhoneDB developers).

### Statistical analysis

Statistical analyses were performed using GraphPad Prism. The data are expressed as the mean ± SEM unless indicated otherwise. Unpaired Student’s *t*-test was used to determine statistically significant differences. A value of *P* < 0.05 was considered significant at the 95% confidence level. Data analysis was performed using the OmicShare tools, a free online platform for data analysis.

### Code availability

The codes generated during this study are available at the OmicShare tools, a free online platform (https://www.omicshare.com/). The software we used is open source software.

## Data availability statement

The datasets produced in this study have been uploaded and are available in the following databases: GSA for Human: Genome Sequence Archive (GSA), with the Accession number: HRA002151 (NGDC - GSA for Human (cncb.ac.cn)).

## Ethics statement

The present study has been reviewed and approved by Sir Run Run Hospital Nanjing Medical University

## Author contributions

L-LY and ZC designed the research, contributed to the execution of the research, analyzed the data and wrote the manuscript. All authors contributed to data analysis, drafting and revising the paper, and agreed to be accountable for all aspects of the work. Y-ZX contributed new reagents or analytic tools. Y-ZX and L-YK reviewed and made significant revisions to the manuscript. All authors contributed to the article and approved the submitted version.

## Funding

This work was supported by the National Natural Science Foundation of China (Grant numbers 81973524); Special funds for science and technology development under the guidance of the central government (ZY20198020 and 2021Szvup163); Key Laboratory of High-Incidence-Tumour Prevention & Treatment (Guangxi Medical University), Ministry of Education (GKE-KF202010); the Drug Innovation Major Project (Grant numbers 2018ZX09711-001-007).

## Conflict of interest

The authors declare that the research was conducted in the absence of any commercial or financial relationships that could be construed as a potential conflict of interest.

## Publisher’s note

All claims expressed in this article are solely those of the authors and do not necessarily represent those of their affiliated organizations, or those of the publisher, the editors and the reviewers. Any product that may be evaluated in this article, or claim that may be made by its manufacturer, is not guaranteed or endorsed by the publisher.
