# Supplementary material for: Single-cell sequencing reveals the landscape of the tumor microenvironment in a skeletal undifferentiated pleomorphic sarcoma patient
Source: Front Immunol. 2022 Nov 16;13:1019870. doi: 10.3389/fimmu.2022.1019870 (PMC9709471; doi:10.3389/fimmu.2022.1019870)
Supplement: Supplementary file 1 [file DataSheet_1.pdf]

Supplementary Material

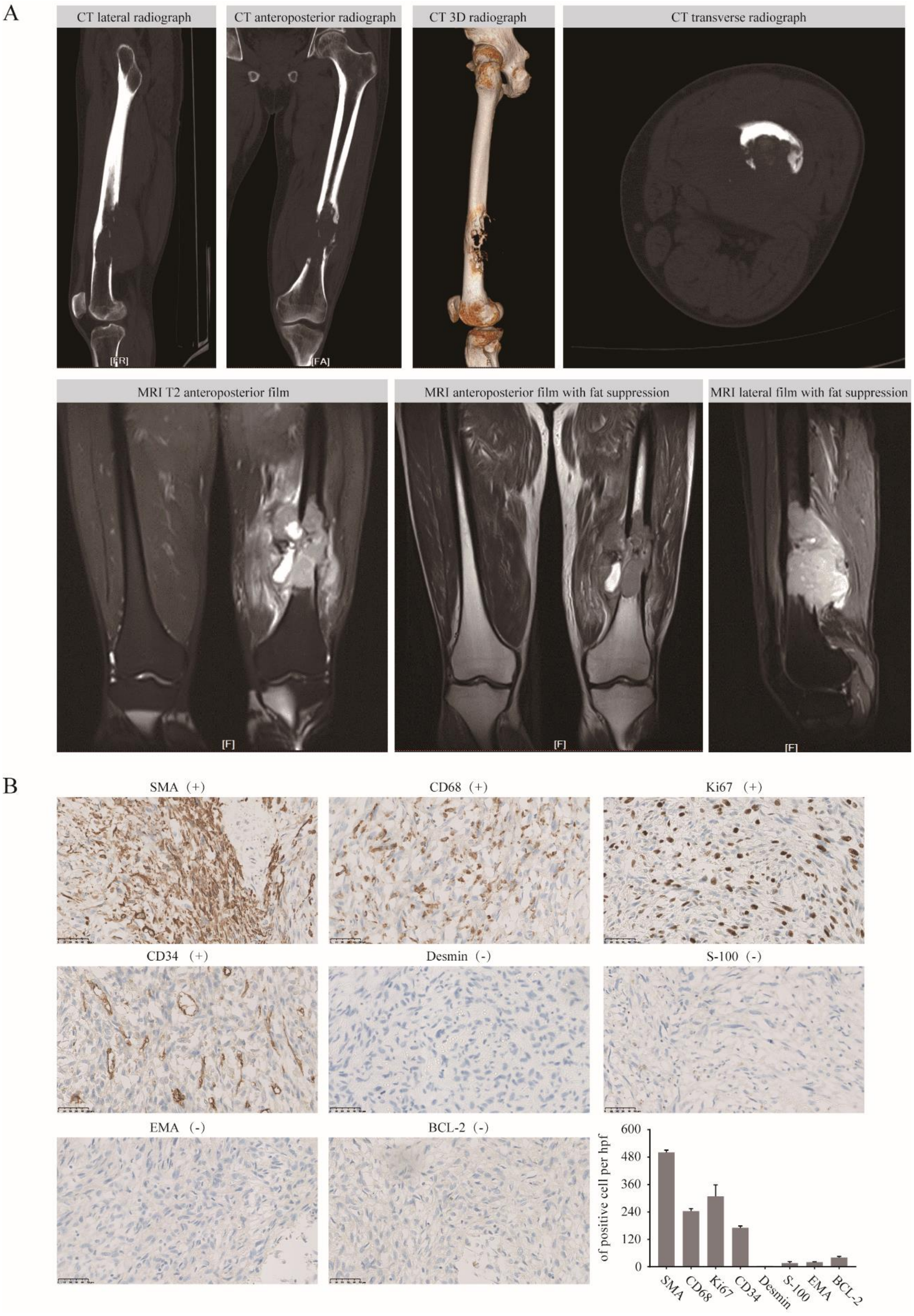

Supplementary Fig. 1 Imaging examinations and further diagnostic workup.

A. Left middle and lower femur occupying space (upper: Computed tomography image; lower: Nuclear Magnetic Resonance Imaging). **B.** Immunohistochemistry staining of SMA, CD68, Ki67, CD34, Desmin, EMA, S-100 and Bcl-2 was performed on tumor sections to validate the type of the tumor sample. Scale bar, 50μm. Quantification of IHC staining from tumor tissues and paracancer tissues (n=3) displayed as the average number of positive cells per high-powered field (×200). Data are shown as the mean ±SEM. *P < 0.05, **P < 0.01, and ***P <0.001.

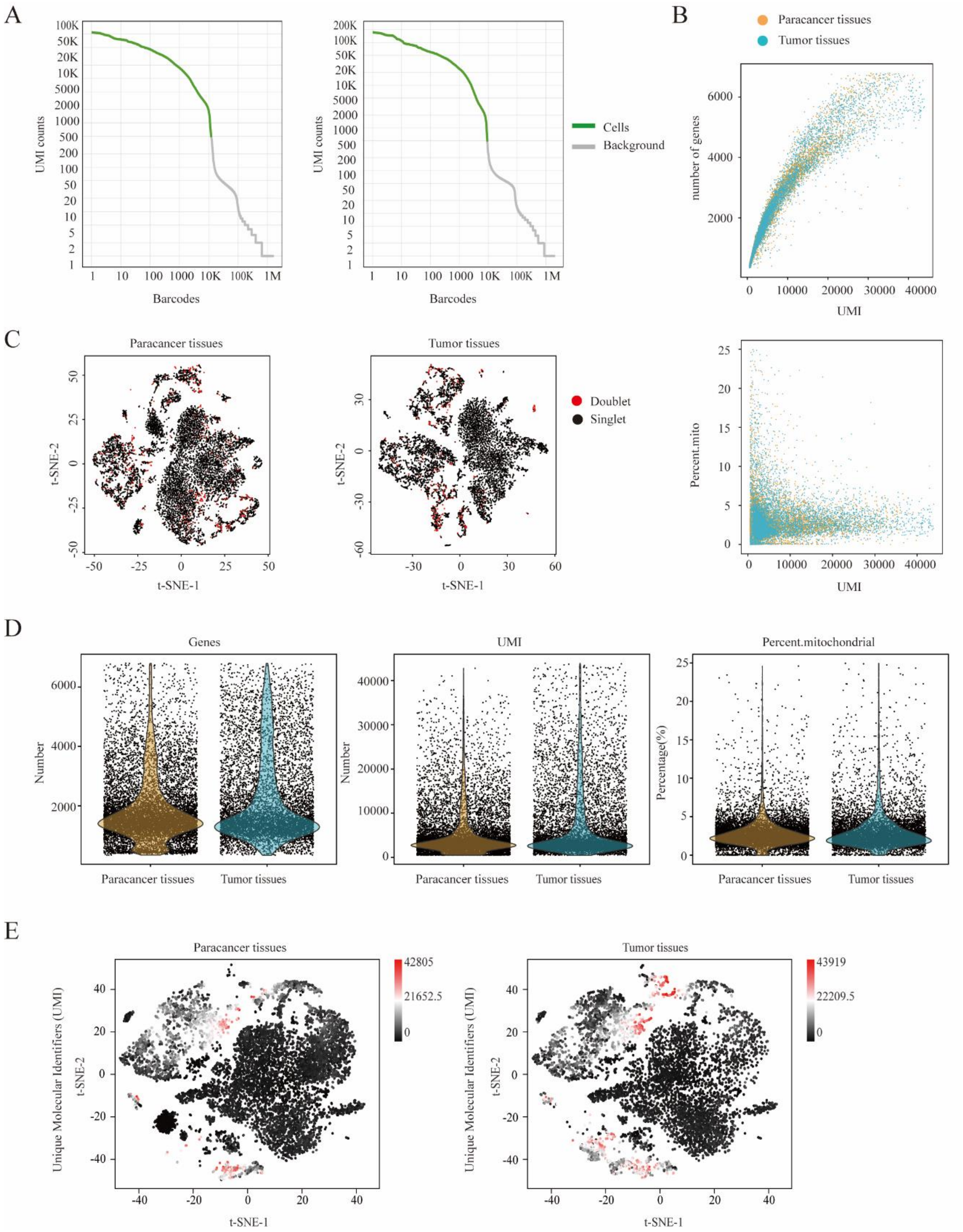

Supplementary Fig. 2 Quality control metrics of scRNA-seq data from SUPS were analyzed.

A. UMI counts of up to standard cells in paracancer tissues (left) and tumor tissues (right). **B.** The percent of mitochondrial genes was shown. **C.** t-SNE plot with the color-coded distribution of doublet and singlet was shown. **D.** Genes/Cell, Unique Molecular Identifiers (UMI)/Cell and percent of

mitochondrial genes/cell genes were shown. **E.** t-SNE plot with color-coded UMIs per cell is shown. Cells with the highest UMI are colored red.

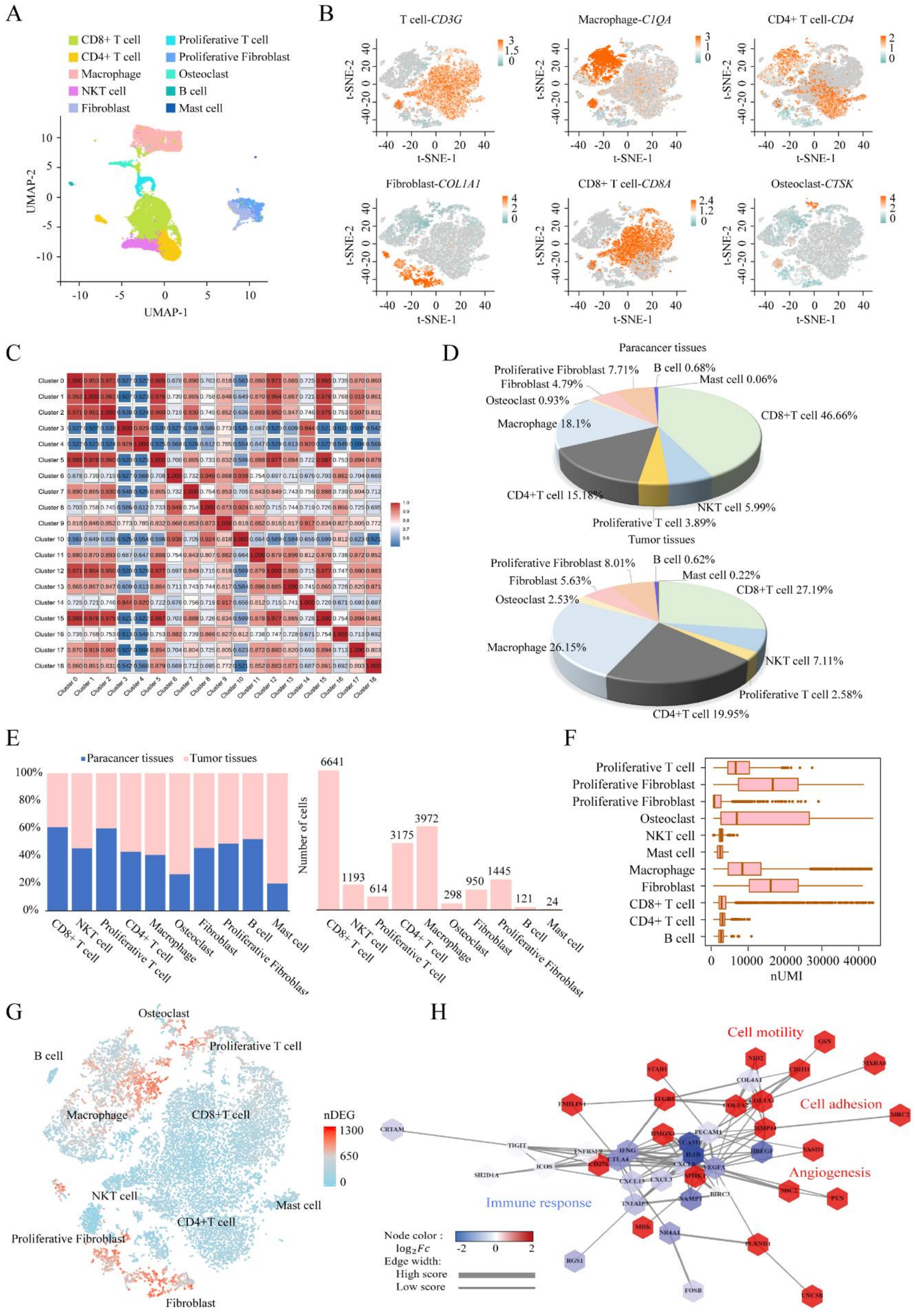

Supplementary Fig. 3 Integrated scRNA-seq analysis of SUPS can identify different cell population characteristics.

A. Uniform manifold approximation and projection (UMAP) plot of the 10 identified main cell types in SUPS lesions, with each cell color-coded according to its associated cell type. **B.** Cell types were identified. Expression of the classical marker genes used to define different cell types. **C.** Similarity analyses of each cell population in paracancer tissues and tumor tissues. **D.** Proportions of all cell populations in tumor tissues and paracancer tissues were shown. **E-F.** For each of the 10 cell clusters, the fraction of cells originating from tumor tissues and paracancer tissues; the number of cells and box plots of the number of transcripts are shown to provide an overview of all cell constitutions. **G.** Number of DEGs between paracancer tissues and tumor tissues within each cluster projected onto the t-SNE map. DEG: |log fold change| > 0.5; adjusted P value <0.05 was derived by a Wilcoxon rank-sum test. **H.** Functional association networks between specific characteristic genes and their expression in tumor tissues compared with paracancer tissues.

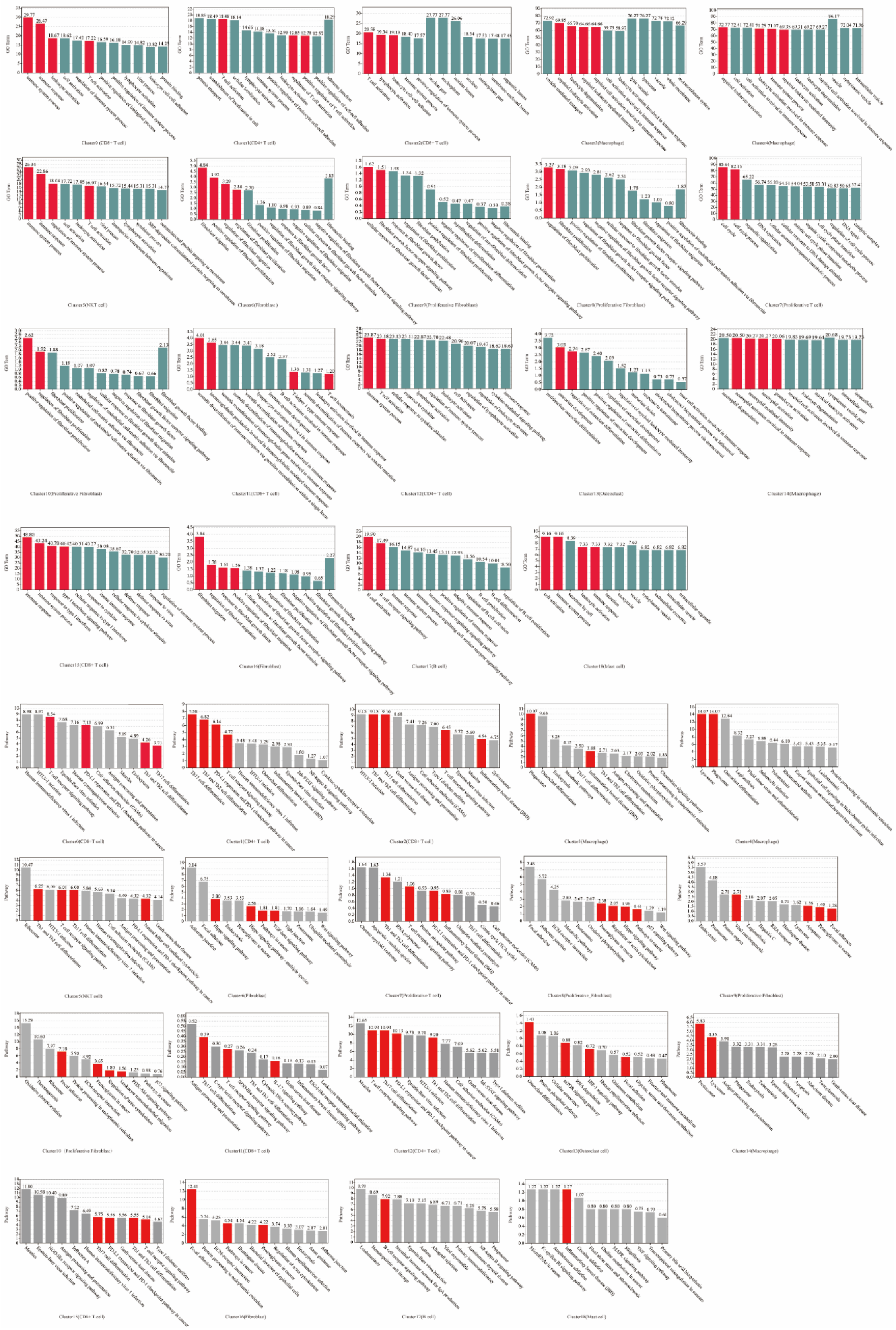

Supplementary Fig. 4 Enriched functions of different subsets by GO and KEGG analyses.

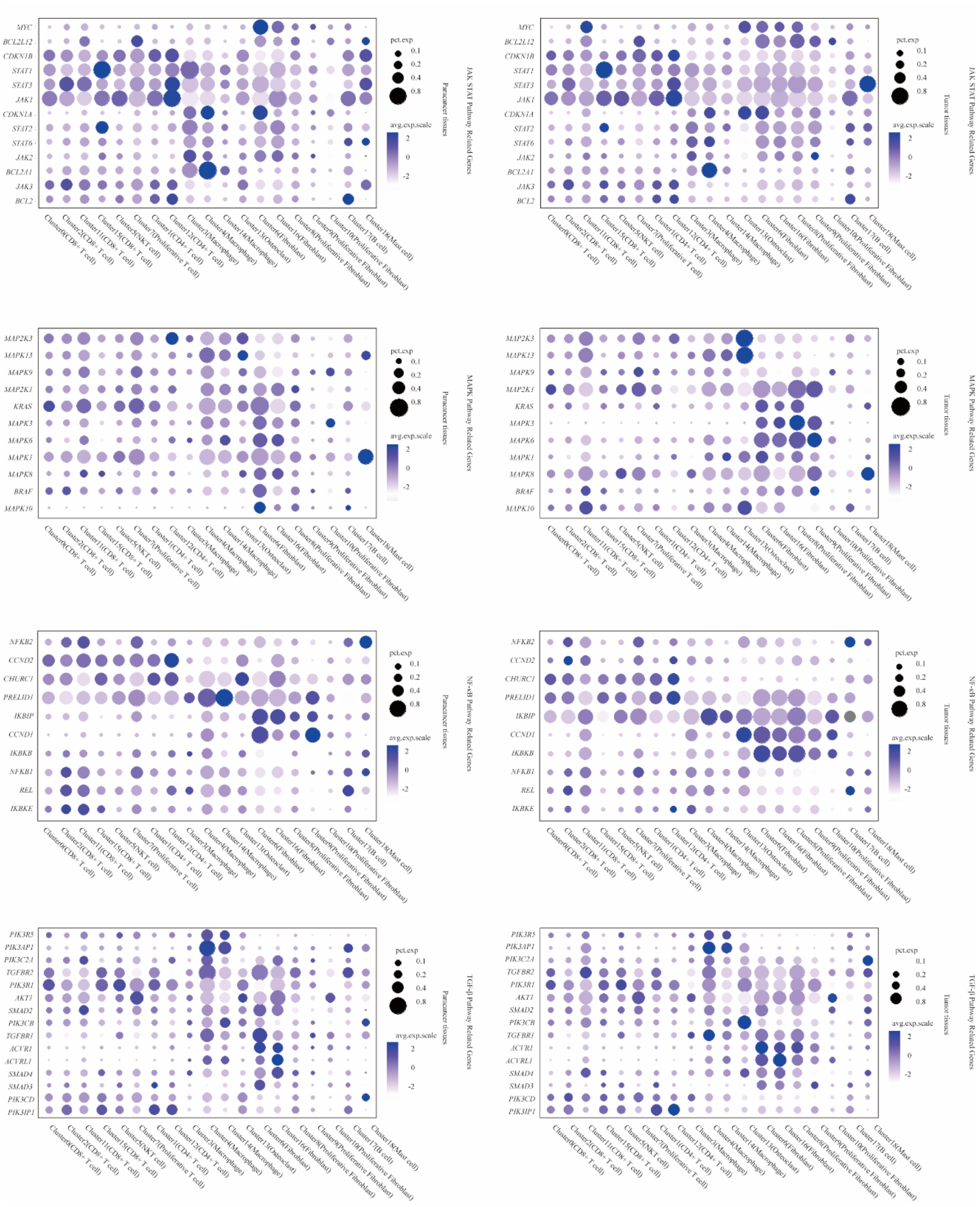

Supplementary Fig. 5 The scRNA-seq data quantified the expression of genes associated with pathways in cancer.

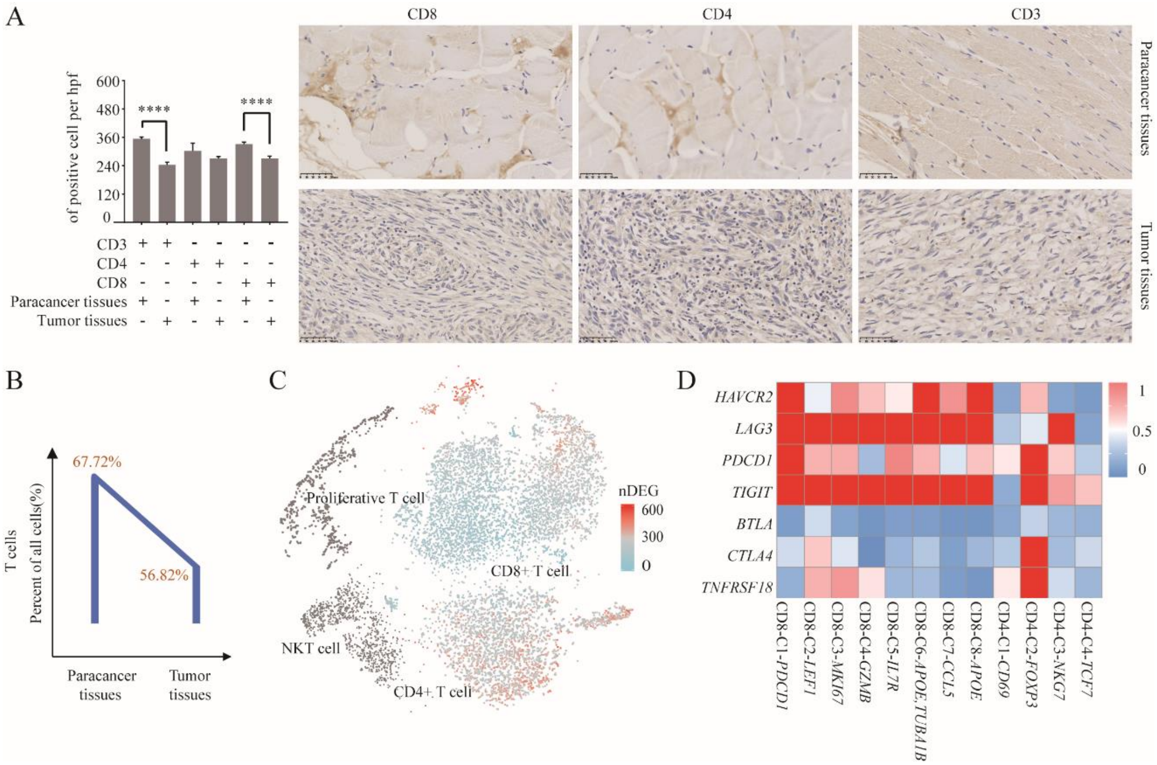

Supplementary Fig. 6 Identify different T cell population characteristics.

A. Immunohistochemistry of SUPS showed the expression of CD3, CD4 and CD8 in paracancer tissues and tumor tissues. Scale bar, 50μm. Quantification of IHC staining from tumor tissues and paracancer tissues (n=3) displayed as the average number of positive cells per high-powered field (×200). Data are shown as the mean ± SEM. **P* < 0.05, ***P* < 0.01, and ****P* <0.001. **B.** The percentages of T cells in paracancer tissues and tumor tissues. **C.** Number of DEGs between paracancer tissues and tumor tissues within T cells projected onto the t-SNE map. **D.** Heatmap showing expression of immune inhibitory molecules for the $CD8^+$ T cells and $CD4^+$ T cells.

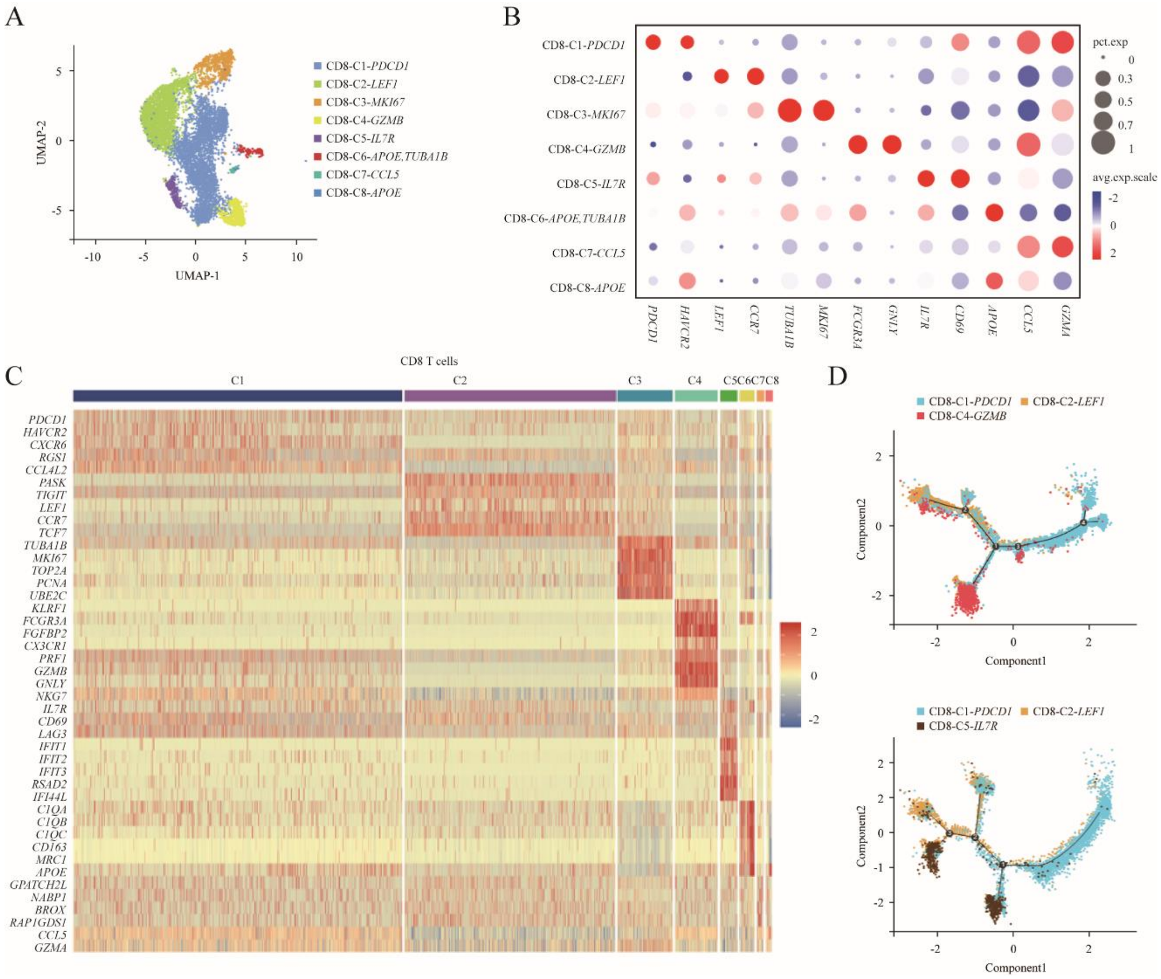

Supplementary Fig. 7 Identify different CD8+ T cell population characteristics.

A. UMAP plot showing the eight main subsets of $CD8^+$ T cells. **B.** Dot plot of specific marker genes in each $CD8^+$ T cell cluster. **C.** Heatmap showing specific marker genes in each $CD8^+$ T cell cluster. **D.** The Monocle 2 trajectory plot showed the dynamics of CD8-C1-*PDCD1*, CD8-C2-*LEF1*, CD8-C4-*GZMB* and CD8-C5-*IL7R*.

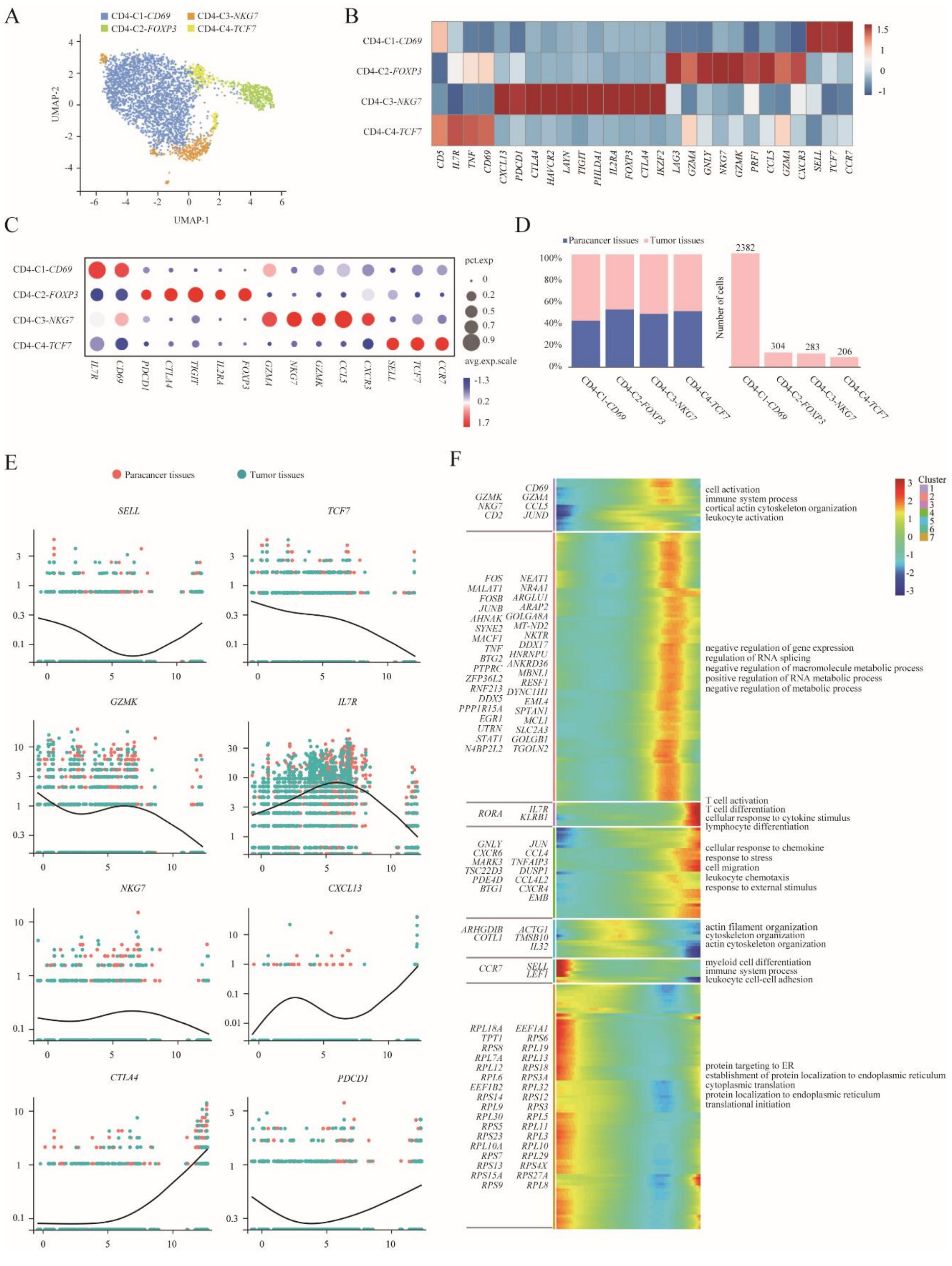

Supplementary Fig. 8 Identify different CD4⁺ T cell population characteristics.

A. UMAP plot showing the four main subsets of $CD4^+$ T cells. **B.** Relative expression map of known marker genes associated with each cell subset. Mean expression values are scaled by mean-centering, and transformed to a scale from -1 to 2. **C.** Dot plot of specific marker genes in each $CD4^+$ T cell cluster. **D.** The cell number and proportion of each $CD4^+$ T cell cluster. **E.** Plot of marker and functional

genes along the CD4$^+$ T cell trajectories. **F.** The DEGs (in rows, q-value < 10−10) in CD4+ T cells (CD4-C2-FOXP3 excluded) along the pseudotime were hierarchically clustered into different subsets. The top annotated GO terms in each cluster were provided.

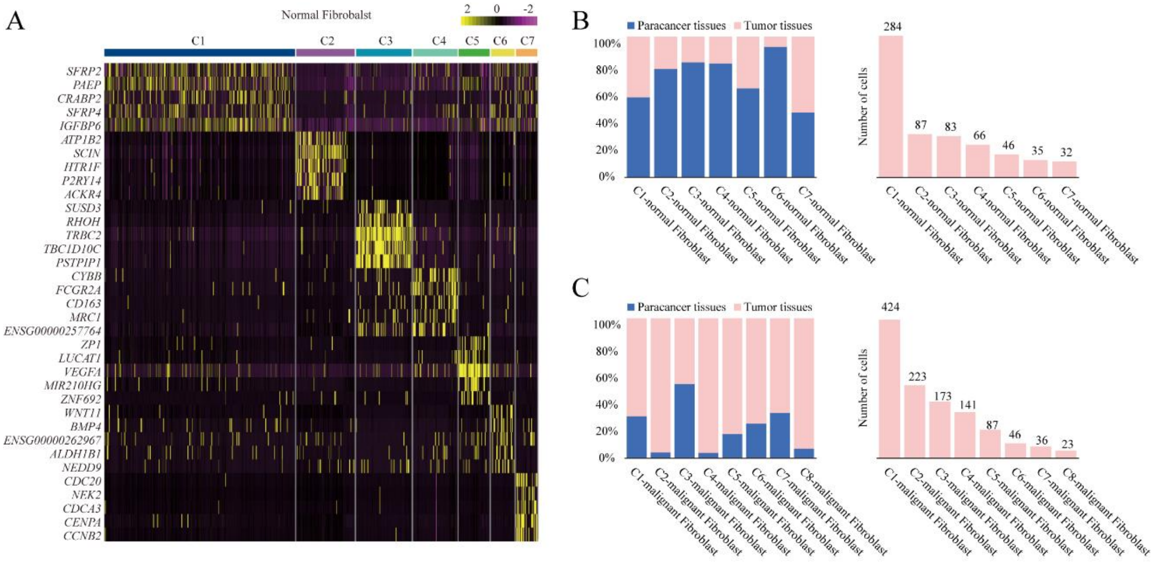

Supplementary Fig. 9 Gene expression heterogeneity of fibroblast subsets was identified in the SUPS.

A. Heatmap showing specific marker genes in each normal fibroblast cluster. **B.** The cell number and proportion of each normal fibroblast cluster. **C.** The cell number and proportion of each malignant fibroblast cluster.
